# Exogenous l-fucose attenuates neuroinflammation induced by lipopolysaccharide

**DOI:** 10.1016/j.jbc.2023.105513

**Published:** 2023-12-01

**Authors:** Xing Xu, Tomohiko Fukuda, Jun Takai, Sayaka Morii, Yuhan Sun, Jianwei Liu, Shiho Ohno, Tomoya Isaji, Yoshiki Yamaguchi, Miyako Nakano, Takashi Moriguchi, Jianguo Gu

**Affiliations:** 1 Division of Regulatory Glycobiology, Institute of Molecular Biomembrane and Glycobiology, Tohoku Medical and Pharmaceutical University, Sendai, Miyagi, Japan; 2 Division of Medical Biochemistry, Tohoku Medical and Pharmaceutical University, Sendai, Miyagi, Japan; 3 Graduate School of Integrated Sciences for Life, Hiroshima University, Higashi-Hiroshima, Japan; 4 Division of Structural Glycobiology, Institute of Molecular Biomembrane and Glycobiology, Tohoku Medical and Pharmaceutical University, Sendai, Miyagi, Japan

**Keywords:** core fucosylation, *N*-glycosylation, microglia, neuroinflammation, interleukin-6

## Abstract

α1,6-Fucosyltransferase (Fut8) catalyzes the transfer of fucose to the innermost GlcNAc residue of *N*-glycan to form core fucosylation. Our previous studies showed that lipopolysaccharide (LPS) treatment highly induced neuroinflammation in Fut8 homozygous KO (Fut8^−/−^) or heterozygous KO (Fut8^+/−^) mice, compared with the WT (Fut8^+/+^) mice. To understand the underlying mechanism, we utilized a sensitive inflammation-monitoring mouse system that contains the human interleukin-6 (*hIL6*) bacterial artificial chromosome transgene modified with luciferase (*Luc*) reporter cassette. We successfully detected LPS-induced neuroinflammation in the central nervous system by exploiting this bacterial artificial chromosome transgenic monitoring system. Then we examined the effects of l-fucose on neuroinflammation in the Fut8^+/−^ mice. The lectin blot and mass spectrometry analysis showed that l-fucose preadministration increased the core fucosylation levels in the Fut8^+/−^ mice. Notably, exogenous l-fucose attenuated the LPS-induced IL-6 mRNA and *Luc* mRNA expression in the cerebral tissues, confirmed using the *hIL6*-*Luc* bioluminescence imaging system. The activation of microglial cells, which provoke neuroinflammatory responses upon LPS stimulation, was inhibited by l-fucose preadministration. l-Fucose also suppressed the downstream intracellular signaling of IL-6, such as the phosphorylation levels of JAK2 (Janus kinase 2), Akt (protein kinase B), and STAT3 (signal transducer and activator of transcription 3). l-Fucose administration increased gp130 core fucosylation levels and decreased the association of gp130 with the IL-6 receptor in Fut8^+/−^ mice, which was further confirmed in BV-2 cells. These results indicate that l-fucose administration ameliorates the LPS-induced neuroinflammation in the Fut8^+/−^ mice, suggesting that core fucosylation plays a vital role in anti-inflammation and that l-fucose is a potential prophylactic compound against neuroinflammation.

Core fucosylation is catalyzed explicitly by α1,6-fucosyltransferase (Fut8) that transfers a fucose residue from GDP-fucose onto the innermost asparagine-linked GlcNAc through an α1,6-linkage in mammals (1). The biosynthesis of core fucosylation demands donor substrate GDP-fucose, which can be synthesized by two distinct pathways: the *de novo* pathway and the salvage pathway (Fig. 1*A*) (2). Under normal conditions, the *de novo* pathway produces up to 90% of GDP-fucose. When this pathway is disrupted, the salvage pathway compensates for the loss of GDP-fucose (3, 4). l-Fucose is a six-deoxy hexose monosaccharide that is abundantly present in plants and seaweed (5, 6). In mammals, free l-fucose originating from dietary sources or the lysosomal catabolism of glycoproteins can directly serve as a substrate for GDP-fucose synthesis *via* the salvage pathway (4, 7). The produced GDP-fucose is subsequently transported into the lumen of the Golgi apparatus through the GDP-fucose transporter and subjected as the donor substrate for fucosyltransferases, such as Fut8, that catalyzes core fucosylation (Fig. 1*A*) (8). Therefore, exogenous l-fucose potentially enhances fucosylation levels. The variety of core fucosylation is intimately involved in the pathophysiological processes of numerous diseases, including pulmonary emphysema (9), schizophrenia (10), cancers such as hepatocellular carcinoma (11), non–small cell lung cancer (12), pancreatic carcinoma (13), and antibody-dependent cellular cytotoxicity (14, 15).

**Figure 1:**
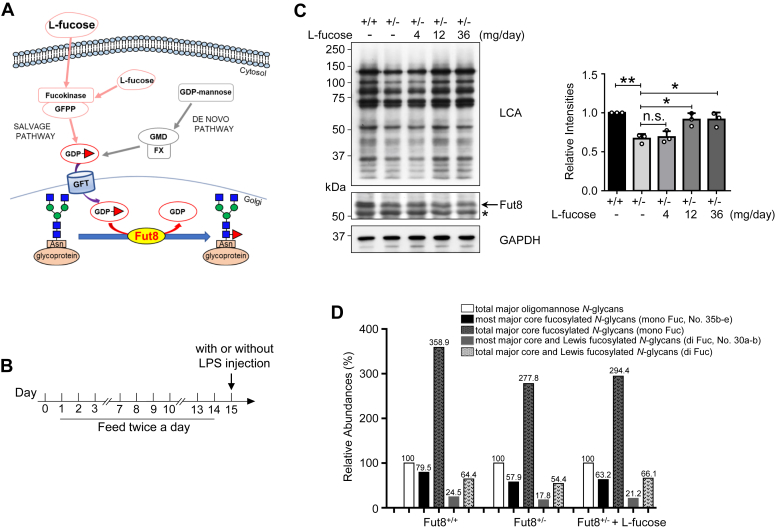
**Effect of exogenous****l****-fucose on core fucosylation in the Fut8**^**+/−**^**mice.***A*, schematic diagram of the salvage and *de novo* pathways for GDP-fucose synthesis. The exogenous l-fucose can be metabolized to the GDP-fucose through the salvage pathway. *B*, schedule of l-fucose administration using gavage twice daily before each experiment (LPS injection in the later experiments). *C*, after the pretreatment described in (*B*), the same amounts of brain tissues were detected by LCA lectin blot and Western blotted with an anti-Fut8 antibody. The *asterisk* indicates the nonspecific band. GAPDH was used as a loading control. The ratio of LCA *versus* GAPDH of Fut8^+/+^ mice treated without l-fucose was set as 1.0. Data were analyzed from all the bands of LCA lectin blot by one-way ANOVA with Tukey's multiple comparison tests and showed as the mean ± SD from three independent experiments. n.s. *p* > 0.05; ∗*p* < 0.05; ∗∗*p* < 0.01. *D*, LC–MS analysis of *N*-glycans obtained from the hippocampus of Fut8^+/+^ mice, Fut8^+/−^ mice, and Fut8^+/−^ mice treated with 36 mg/day l-fucose. The intensities of major oligomannose *N*-glycans (nos.: 2–5), most major core fucosylated *N*-glycans (mono Fuc, no. 35b–e), major core fucosylated *N*-glycans (mono Fuc, nos.: 10, 11, 14a, 17b,c, 20b, 21, 26c, 27, 29b,c, 30a,b, 32c, 33, 35b–e, 36a,b, 39b, 41a,c, 42a, 43a, 45b, 46b,c, 49a,b, 50a, 53a, 54a, 57, 58, based on diagnostic ion in MS/MS), most major core and Lewis fucosylated *N*-glycans (di Fuc, no.: 30a,b), and major core and Lewis fucosylated *N*-glycans (di Fuc, nos.: 18, 21, 27, 30a,b, 36b, 42a, 43a, 54a, 58 based on diagnostic ion in MS/MS) are shown in Fig. S1*D*. The relative abundances (%) calculated by setting the total intensities of major oligomannose *N*-glycans as 100%. Each data were obtained from a mixture of three mice. *LCA*, *Lens culinaris* agglutinin; LPS, lipopolysaccharide.

It has been well established that neuroinflammation is a normal immune response resulting from numerous pathological injuries, including ischemia, toxins, infection, and trauma in the central nervous system (CNS) (16). Neuroinflammation cascades primarily depend on the activation of microglial cells, which are the CNS-resident innate immune cells stemming from the yolk sac macrophages (17, 18, 19). In addition, upon pathological stimuli or neuronal insults, microglia secrete proper concentrations of immune mediators, that is, interleukin-6 (IL-6), IL-1β, nitric oxide, and tumor necrosis factor-alpha to coordinate neuroinflammation (19, 20). Among numerous proinflammatory cytokines, IL-6 is the most pivotal cytokine for acute-phase responses (14). It can be produced by various types of cells, including microglia, astrocytes, neurons, macrophages, and B cells (21, 22). IL-6 is a multifunctional and pleiotropic cytokine and is associated with multiple sclerosis (23), experimental autoimmune encephalomyelitis (24), perioperative neurocognitive disorders (25), and depression (26). In the CNS, proinflammatory IL-6 signaling is mainly mediated *via* trans-signaling with soluble IL-6 receptor (sIL-6R) that subsequently constitutes a complex with glycoprotein 130 kDa (gp130) and activates downstream Janus kinase (JAK)/signal transducer and activator of transcription (STAT) signaling pathway (27, 28). Previous studies have confirmed that many types of cytokine and immune receptors, such as transforming growth factor beta 1 receptor (9), B-cell receptor (15), T-cell receptor (15, 29), integrin α3β1 (30), and epidermal growth factor receptor (31), contain core fucosylation that differently regulates their biological functions.

Fut8 homozygous KO (Fut8^−/−^) mice exhibit a schizophrenia-like phenotype with a decrease in working memory (10) and long-term potentiation (32). Curiously, patients suffered from a complete loss of core fucosylation because of biallelic Fut8 mutations (33) that showed growth retardation, and severe developmental and neurological impairment, which were quite similar to the abnormal phenotypes observed in the Fut8^−/−^ mice (9, 10). While the pathophysiology of schizophrenia has not yet been fully elucidated, several studies suggest that neuroinflammation leading to glial dysfunction could contribute to the pathogenesis of schizophrenia (34, 35). Our previous study revealed that core fucosylation negatively regulated the sensitivity of glia to inflammatory stimuli (36). The initial activation status of glial cells in the neuroinflammation induced by lipopolysaccharide (LPS) was significantly enhanced in the Fut8^−/−^ mice, compared with WT (Fut8^+/+^) mice. Notably, the degree of neuroinflammation in the Fut8 heterozygous KO (Fut8^+/−^) mice ranged between that of Fut8^+/+^ and Fut8^−/−^ mice, presumably because of haploinsufficiency of Fut8. Consistently, the Fut8^+/−^ mice exhibited greater sensitivity to cigarette smoke–induced emphysema than the Fut8^+/+^ mice (37).

In the present study, we explore the importance of core fucosylation and its participation in neuroinflammation, utilizing the bacterial artificial chromosome (BAC)–based human IL-6 gene (*hIL6*)–driven firefly luciferase reporter transgenic mice that we previously generated (21). After crossbreeding the *hIL6*-BAC-*Luc* mice with the Fut8^+/−^ mice, we obtained the Fut8::*hIL6*-*Luc* compound transgenic mice. Using this mouse strain, we quantitatively monitored the neuroinflammation induced by LPS. We found that the Fut8^+/−^::*hIL6*-*Luc* mice showed a higher neuroinflammatory response than Fut8^+/+^::*hIL6*-*Luc* transgenic mice. Exogenous l-fucose ameliorated the LPS-induced neuroinflammatory responses, including glial cell activation and several cytokine expressions in the Fut8^+/−^ mice. Considering that l-fucose is a natural and nontoxic food ingredient, such as seaweed (38), the present study proposes its potential utility for the treatment or prevention of neuroinflammation.

## Results

### Exogenous l-fucose increased the core fucosylation in brain tissues of Fut8^+/−^ mice

It has been known that the Fut8^+/−^ mice exhibit a reduced amount of Fut8 and consequently diminish the core fucosylation level compared with the Fut8^+/+^ control mice. To investigate whether exogenous l-fucose improves the core fucosylation level in Fut8^+/−^ mice, we fed the mice by oral gavage with different doses of l-fucose twice a day for 2 weeks, as shown in Figure 1*B*. Expectedly, the expression levels of core fucosylation in brain tissues detected by lectin blotting with *Lens culinaris* agglutinin (LCA), which preferentially recognizes core fucose (39), were lower in Fut8^+/−^ mice than in Fut8^+/+^ mice (Fig. 1*C*). Similarly, the expression levels of Fut8 protein were also lower in Fut8^+/−^ mice (Fig. 1*C*). The decreased levels of core fucosylation in the Fut8^+/−^ mice were significantly rescued by l-fucose administration at 12 or 36 mg/day (Fig. 1*C*). Consistently, the results obtained from mass spectrometry (MS) analysis (Fig. S1) also showed that the levels of major *N*-glycans containing core fucose in the hippocampus of Fut8^+/−^ mice were lower than that in Fut8^+/+^ mice (Fig. 1*D*). As anticipated, the decreased levels of core fucosylation in the hippocampus were partially rescued by exogenous l-fucose (Fig. 1*D*). Furthermore, HPLC separation of nucleotide sugars demonstrated that the levels of GDP-fucose increased after l-fucose administration at 12 or 36 mg/day (Fig. S2). Surprisingly, the level of GDP-fucose was decreased in the Fut8^+/−^ mice compared with in Fut8^+/+^ mice. These results demonstrate that exogenous l-fucose can increase the modification of core fucosylation in the brain tissues *in vivo*.

### Exogenous l-fucose attenuated the neuroinflammation monitored by the luciferase luminescence *via* the WIM-6 system

**Figure 2:**
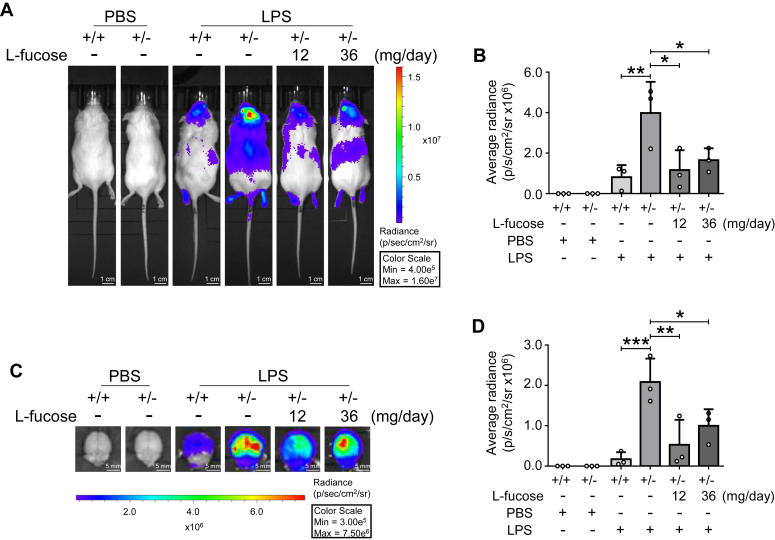
**Monitoring the neuroinflammation by the WIM-6 system.***A*, representative images of luciferase luminescence *in vivo* 4 h after injection with or without LPS (1 mg/kg) in different groups were indicated. *B*, quantitative analysis of intensities for luciferase luminescence. All data were analyzed by one-way ANOVA test and shown as the mean ± SD from three independent experiments. The *ex vivo* imaging system also examined luciferase luminescence induced by LPS. *C*, representative images of luciferase luminescence in brain tissues. *D*, quantitative analysis of intensities for luciferase luminescence *ex vivo*. All data were shown as the mean ± SD from three independent experiments. ∗*p* < 0.05; ∗∗*p* < 0.01; ∗∗∗*p* < 0.001. LPS, lipopolysaccharide.

Next, we evaluated the therapeutic efficacy of l-fucose against neuroinflammation *via* the inflammation-monitoring system, namely whole-body *in vivo* monitoring employing the *hIL6*-BAC-*Luc* transgenic system (WIM-6 system), which can evaluate different levels of inflammatory responses by examining the intensities of luciferase luminescence (21). The luciferase luminescence was analyzed using *in vivo* imaging system (IVIS) 4 h after the LPS treatment. The IVIS results showed that the luciferase luminescence in brain tissues was markedly increased in Fut8^+/−^::*hIL6*-*Luc* mice than that of Fut8^+/+^::*hIL6*-*Luc* mice (Fig. 2, *A* and *B*). Very interestingly, the 14-day constant l-fucose pretreatment (Fig. 1*B*) dramatically reduced the bioluminescence (Fig. 2, *A* and *B*). Subsequently, we also performed an *ex vivo* imaging to ask whether l-fucose diminishes the LPS-induced neuroinflammation. Consistent with the IVIS results, the *ex vivo* results showed a partial reduction of the *hIL6*-*Luc* luminescence upon l-fucose administration (Fig. 2, *C* and *D*). These results (Fig. 2, *C* and *D*) suggest that the effects of l-fucose were nondose responsive. The exact reason for this phenomenon needs further clarification. We hypothesize that Fut8 may preferentially utilize the GDP-fucose originating from the exogenous l-fucose (40), the 12 mg/day l-fucose dosage might reach a saturation effect. In contrast, the 36 mg/day l-fucose dosage produced more GDP-fucose (Fig. S2), potentially exceeding the capacity for GDP-fucose utilization by Fut8 enzyme in Fut8^+/−^ mice. This surplus could be utilized by other fucosyltransferases to modify *N*-glycan antennae. In addition, a higher dose of l-fucose might alter the sugar metabolism. These together may lead to some unexpected outcomes. The underlying mechanisms require further elucidation. These results indicate that l-fucose can ameliorate the neuroinflammation induced by LPS.

### Effects of exogenous l-fucose on neuroinflammation induced by LPS

**Figure 3:**
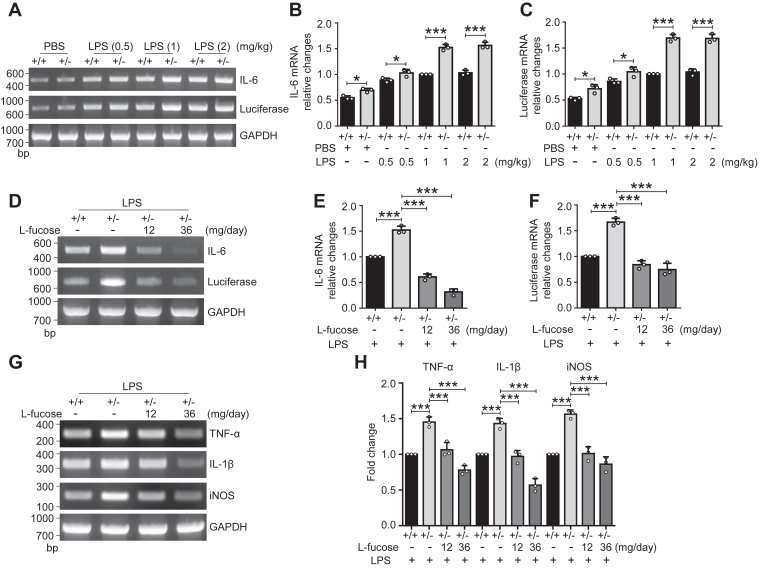
**Alteration of inflammatory cytokines induced by LPS.** The Fut8::*hIL6*-*Luc* mice were treated with equal amounts of PBS or LPS at indicated concentrations *via* intraperitoneal injection. Post 4 h after intraperitoneal injection, RT–PCR (*A*) and real-time PCR (*B* and *C*) detected the mRNA levels of IL-6 and luciferase in the brain tissues. The ratio of IL-6 or luciferase *versus* GAPDH of Fut8^+/+^::*hIL6*-*Luc* mice injected with 1 mg/kg LPS was set as 1.0. Values were shown as the mean ± SD from three independent experiments (one-way ANOVA test). ∗*p* < 0.05; ∗∗∗*p* < 0.001. *D*–*H*, the Fut8::*hIL6*-*Luc* mice were treated with equal amounts of PBS or l-fucose (12 or 36 mg/day) for 2 weeks and then intraperitoneally injected with LPS on the 15th day. Post 4 h after intraperitoneal injection, RT–PCR (*D* and *G*) and real-time PCR (*E*, *F*, and *H*) detected the levels of inflammation factors, including IL-6 and luciferase, tumor necrosis factor-alpha (TNF-α), IL-1β, and inducible nitric oxide synthase (iNOS) in the brain tissues. GAPDH was used as an internal control. Each value was normalized to that of the GAPDH. The value of Fut8^+/+^::*hIL6*-*Luc* mice treated without l-fucose was set as 1.0. All data were shown as the mean ± SD from three independent experiments. ∗∗∗*p* < 0.001. hIL6, human IL-6 gene; IL, interleukin; LPS, lipopolysaccharide.

To further explore the efficacy of l-fucose administration on neuroinflammation, we examined expression levels of several cytokines and mediators related to inflammation. We administered the different doses (0.5, 1, and 2 mg/kg) of LPS as an inflammatory stimulus to the Fut8::*hIL6*-*Luc* mice. RT–PCR and real-time PCR assays revealed that the LPS administration induced the IL-6 and luciferase mRNA expression dose-dependently in the Fut8^+/−^ and Fut8^+/+^ mice (Fig. 3, *A*–*C*). The induced IL-6 and luciferase mRNA expression were significantly higher in the Fut8^+/−^ mice than in the Fut8^+/+^ mice (Fig. 3, *A*–*C*). Of note, the basal mRNA expression of IL-6 and luciferase in the absence of the LPS stimulus was higher in the Fut8^+/−^ mice than in the Fut8^+/+^ mice (Fig. 3, *A*–*C*). This result is consistent with the previous observation that Fut8^−/−^ mice showed a spontaneous increase in microglial activation *in vivo* (36). In the subsequent experiments, we selected the dose at 1 mg/kg of LPS as an inducer of the neuroinflammatory model since both 1 and 2 mg/kg of LPS induced significantly different levels of IL-6 and luciferase mRNA between Fut8^+/−^ and Fut8^+/+^ mice.

Since exogenous l-fucose rescued the core fucosylation level and neuroinflammation in the cerebral tissues of Fut8^+/−^ mice, as described previously, we examined whether l-fucose administration could alleviate the inflammatory responses induced by LPS. Consistent with the IVIS results, the RT–PCR and real-time PCR results showed that the induction of IL-6 and luciferase expression by LPS were much higher in the Fut8^+/−^ mice than in the Fut8^+/+^ mice. The induction was significantly suppressed by l-fucose administration (Fig. 3, *D*–*F*). The results in Figure 3*F* did not align with the luciferin signals in Figure 2. This discrepancy could be attributed to two factors (1) the nonlinear correlation between the expression levels of luciferase and luciferin signals because luciferase is an enzyme and (2) a compromised blood–brain barrier in Fut8^+/−^ mice, potentially leading to improved penetration of LPS and/or luciferin into brain parenchyma, since the deficiency of Fut8 may suppress the expression of vascular endothelial growth factor receptor-2 and subsequently affect angiogenesis (41). Further investigation is needed to elucidate the detailed mechanisms. Given that LPS injection may also increase proinflammatory markers, we further assessed mRNA expression levels of tumor necrosis factor-alpha, IL-1β, and inducible nitric oxide synthase in the brain tissues. Again, the results showed that the expression levels of these proinflammatory cytokines were higher in the Fut8^+/−^ mice than in the Fut8^+/+^ mice. The induction by LPS was significantly suppressed in an l-fucose dose-dependent manner (Fig. 3, *G* and *H*). These results demonstrate that a lower core fucosylation leads to enhanced neuroinflammatory status and higher sensitivity to inflammatory stimulators, which can be corrected by exogenous l-fucose administration.

### l-fucose inhibited microglia activation induced by LPS in the dentate gyrus

**Figure 4:**
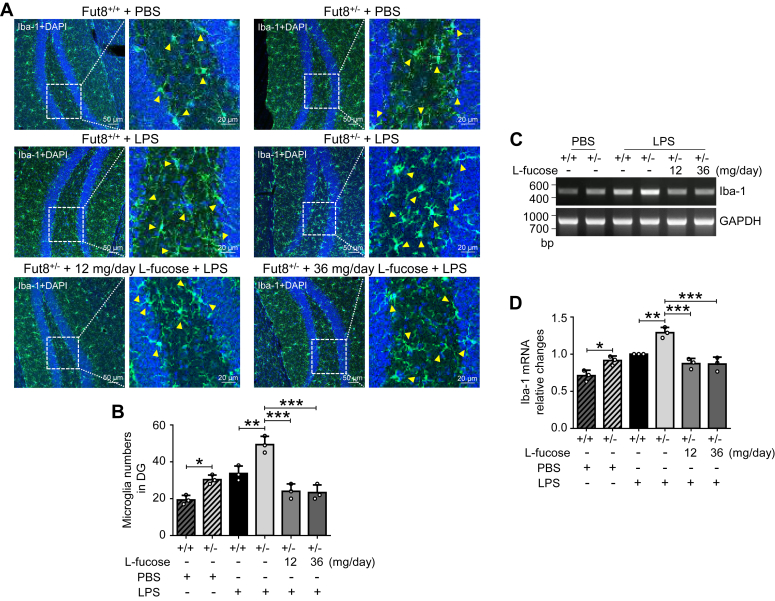
**The overactivation of microglia induced by LPS was inhibited after****l****-fucose pretreatment.** The Fut8::*hIL6*-*Luc* mice were treated with equal amounts of PBS or l-fucose twice a day for 2 weeks and then intraperitoneally injected with PBS or LPS for 4 h on the 15th day, as described for Figure 1*B*. *A*, representative immunostaining images with anti-Iba1 antibody and DAPI in the DG of brain tissues. *Arrows* indicate the activated microglia. *B*, quantitative analysis of the activated microglia. All data were shown as the mean ± SD from three independent experiments (one-way ANOVA test). ∗*p* < 0.05; ∗∗*p* < 0.01; ∗∗∗*p* < 0.001. The expression levels of Iba-1 mRNA in the DG of brain tissues were further detected by RT–PCR (*C*) and real-time PCR (*D*). The ratio of Iba-1 *versus* GAPDH of Fut8^+/+^::*hIL6*-*Luc* mice treated without l-fucose and injected with LPS was set as 1.0. All data for the quantitative analysis of the changes were shown as the mean ± SD from three independent experiments. ∗*p* < 0.05; ∗∗*p* < 0.01; ∗∗∗*p* < 0.001. DAPI, 4′,6-diamidino-2-phenylindole; DG, dentate gyrus; *hIL6*, human IL-6 gene; LPS, lipopolysaccharide.

Microglia, which account for approximately 10% of brain cells, play a pivotal role in active immune defense (42, 43). Upon pathogen invasion or inflammatory stimuli, microglia transit to an activated state and generate inflammatory mediators to participate in the immune response and debris clearance (42). Nevertheless, if the stimulation exists persistently, the activated microglial cells would cause irreparable CNS injury and neuroinflammation-associated psychiatric disorders (20, 42, 44). One aspect of dendate gyrus physiology is that it can generate new neurons throughout life (45); meanwhile, aberrant microglial activation in the dentate gyrus can impair neurogenesis and cell survival (46) and lead to depression-like neurological symptoms (43, 47). Therefore, suppressing microglial overactivation can be a potential strategy for preventing psychiatric diseases. Given these, we examined the glial cell activation status by detecting ionized calcium-binding adaptor molecule-1 (Iba-1), a microglia marker, in the hippocampus regions. The immunostaining results showed that the luciferase was highly expressed in microglia (Fig. S3). Furthermore, the immunostaining with anti-Iba-1 antibody showed a significant difference between Fut8^+/−^ and Fut8^+/+^ mice under normal conditions without LPS treatment, that is, more Iba-1-positive cells in the Fut8^+/−^ mice (Fig. 4, *A* and *B*). On the other hand, after treatment with LPS, the increase in Iba-1-positive cells was more significant in the Fut8^+/−^ mice than in the Fut8^+/+^ mice. These enhanced staining and activation were dramatically rescued by exogenous l-fucose (Fig. 4, *A* and *B*). Consistently, the RT–PCR and real-time PCR results also confirmed that the expression levels of Iba-1 were higher in Fut8^+/−^ mice than in the Fut8^+/+^ mice, which were suppressed by l-fucose (Fig. 4, *C* and *D*). These results further suggest that core fucosylation is crucial for maintaining normal microglial status and that l-fucose supplementation can alleviate the aberrantly activated microglia.

### Fut8 deletion enhanced the association of gp130 with IL-6R and decreased the effects of l-fucose on IL-6 expression

**Figure 5:**
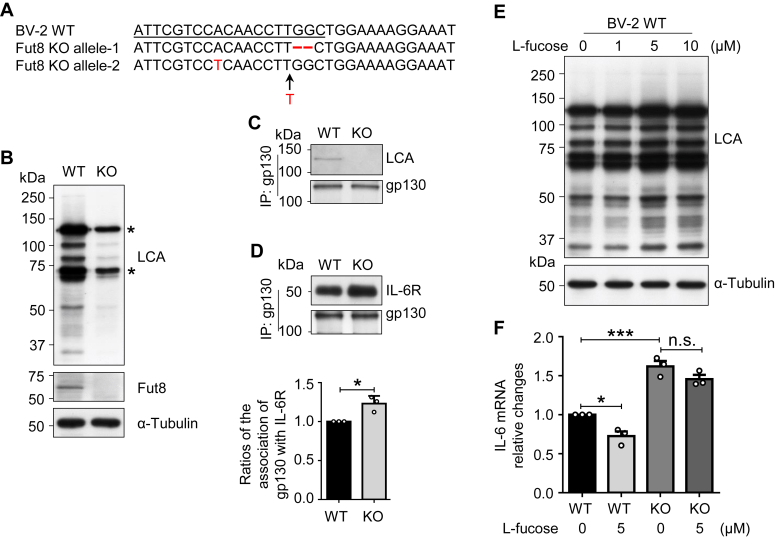
**Fut8 negatively regulated the interaction between gp130 and IL-6R and decreased the effects of****l****-fucose on IL-6 expression.***A*, the generation of the Fut8-KO cell line was described in the Experimental procedures section and confirmed *via* genomic sequence analysis. *B*, the validation of Fut8-KO was performed using lectin blotting with LCA and Western blotting using indicated antibodies. *Asterisks* indicate nonspecific staining. *C*, equal amounts of cell proteins were immunoprecipitated using Ab-Capcher with an anti-gp130 antibody. The immunoprecipitated samples were subjected to lectin blotting using LCA lectin. *D*, post 4 h after LPS (1000 ng/ml) pretreatment, equal amounts of cell lysates were immunoprecipitated with an anti-gp130 antibody. Then the immunoprecipitants were Western blotted with the indicated antibodies. Data were quantified by ImageJ software and were shown as the mean ± SD from three independent experiments. The ratio of IL-6R *versus* gp130 of WT cells was set as 1.0. ∗*p* < 0.05 (unpaired Student's *t* test). *E*, the WT cells were cultured with l-fucose for 24 h at the indicated concentrations. Equal amounts of cell lysates were detected by LCA lectin, and α-tubulin was used as a loading control. *F*, the cells were pretreated with or without l-fucose at 5 μM for 24 h and subsequently stimulated with LPS for 4 h. The mRNA expression levels of IL-6 were detected by real-time PCR. GAPDH was used as an internal control. Each value was normalized to that of the GAPDH. The value of WT cells treated without l-fucose was set as 1.0. Data represent the mean ± SD from three independent experiments. n.s. *p* > 0.05; ∗*p* < 0.05; ∗∗∗*p* < 0.001 (one-way ANOVA test). IL, interleukin; LCA, dentate gyrus.

It has been known that the proinflammatory property of IL-6 is mediated predominantly through the trans-signaling (27, 48). In the trans-signaling pathway, IL-6 binds to the sIL-6R, forming the IL-6–sIL-6R complex, which interacts with gp130. A series of previous studies have demonstrated that core fucosylation can either positively or negatively regulate the function of the cell surface receptors. For example, core fucosylation of epidermal growth factor receptor is required for its higher affinity with epidermal growth factor ligand to upregulate downstream signaling (31), and core fucosylation on folate receptor α (FOLR1) strengthens the uptake capacity of folate (49). Conversely, the core fucosylation negatively regulates some receptors and their downstream signaling, such as α-amino-3-hydroxy-5-methyl-4-isoxazolepropionic acid receptor and activin receptor (32, 50), as well as FcγRIII receptor (51). Considering gp130 is the most critical coreceptor for IL-6, which can mediate the downstream JAK–STAT signaling pathway (27, 28), we hypothesized that the core fucosylation might regulate the complex formation between IL-6R and gp130. We used the CRISPR–Cas9 system to establish the Fut8 KO BV-2 cell line, confirmed by genomic sequence analysis. The analysis revealed a 2-base (GG) deletion in allele 1, one mutation (A was replaced by T in the red) in allele 2, and one insertion mutation (T inserted between T and G) compared with the Fut8 WT cells (Fig. 5*A*). Furthermore, the results from LCA lectin blotting and Western blot using anti-Fut8 antibody confirmed the successful deletion of the Fut8 gene (Fig. 5*B*). The core fucosylation of gp130 was abolished in the Fut8-KO cells (Fig. 5*C*). Subsequently, we validated the complex formation between IL-6R and gp130 through coimmunoprecipitation experiments. These experiments demonstrated a significant increase in the association between gp130 and IL-6R in the Fut8-KO cells compared with that in the WT cells (Fig. 5*D*). In addition, we validated the “feed-forward” mechanism using the BV-2 cell line. The LCA lectin results showed that the levels of core fucosylation were increased after l-fucose pretreatment at different doses (Fig. 5*E*), in which the dose at 5 μM shows sufficient effect on the increase. The real-time PCR assay showed that the expression levels of IL-6 were increased in the Fut8-KO cells, compared with that in the WT cells. Interestingly, the induction of IL-6 was suppressed by the l-fucose supplementation in the WT cells, whereas the enhanced expression levels of IL-6 in the Fut8-KO cells could not be suppressed by the l-fucose (Fig. 5*F*). These findings strongly suggest the notion that core fucosylation negatively regulates the complex formation between gp130 and IL-6R.

To gain structural insights on the core fucosylation on gp130, we built a 3D structural model of *N*-glycosylated IL-6–sIL-6R–gp130 complex based on the cryo-EM structure of human IL-6–sIL-6R/–gp130 complex (Fig. S4) (52). Gp130 is heavily glycosylated, especially *N*-glycans on N43, N61, N83, N131, and N157, which are clustered in close proximity with the other gp130 and IL-6 molecules. Furthermore, four *N*-glycans (N131 and N225 on gp130) occupy the interior space between two gp130 molecules. It is possible that core fucosylation modulates the relative orientation of the attached *N*-glycans with respect to the polypeptide and then modulates the protein–protein interactions. Further analysis is required in the future for a better understanding of the core fucosylation on gp130, such as kinetic interaction study.

### Effects of exogenous l-fucose on the gp130–JAK2–Akt–STAT3 signaling

**Figure 6:**
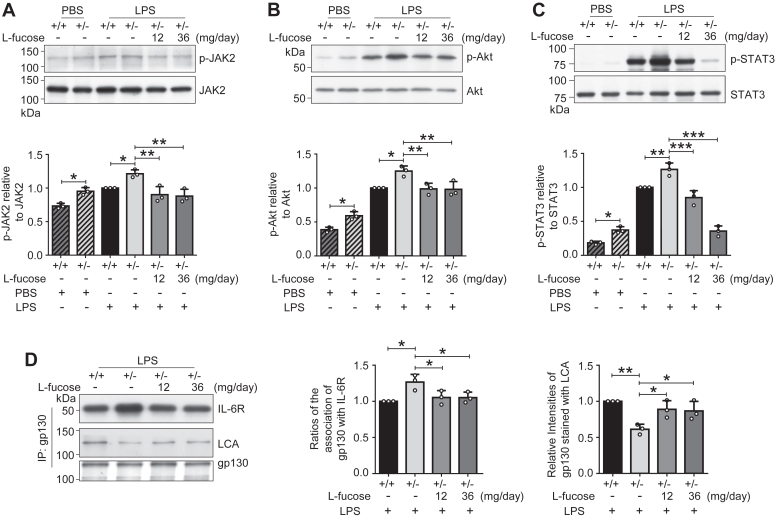
**Effects of core fucosylation on intracellular signaling.** The brain tissues were obtained from the pretreated Fut8::*hIL6*-*Luc* mice as described for Figure 1*B*. The expression levels of phosphor-JAK2 (p-JAK2) and JAK2 (*A*), phosphor-Akt (p-Akt) and Akt (*B*), phosphor-STAT3 (p-STAT3) and STAT3 (*C*) were examined by Western blotting with the indicated antibodies. Data were quantified by ImageJ software. The ratios of p-JAK2 *versus* JAK2, p-Akt *versus* Akt, or p-STAT3 *versus* STAT3 of Fut8^+/+^::*h*IL6-*Luc* mice treated without l-fucose and injected with LPS were set as 1.0. Data were shown as the mean ± SD from three independent experiments. ∗*p* < 0.05; ∗∗*p* < 0.01; ∗∗∗*p* < 0.001 (one-way ANOVA test). *D*, equal amounts of tissue proteins were immunoprecipitated with Ab-Capcher combined with anti-gp130 antibody, and the immunoprecipitates were lectin blotted with LCA lectin or Western blotted with anti-IL-6R and anti-gp130 antibodies. Data were quantified by ImageJ software. The ratio of IL-6R or LCA *versus* gp130 of Fut8^+/+^::*hIL6*-*Luc* mice treated without l-fucose was set as 1.0. All data were shown as the mean ± SD from three independent experiments. ∗*p* < 0.05; ∗∗*p* < 0.01 (one-way ANOVA test). Akt, protein kinase B; *hIL6*, human IL-6 gene; IL, interleukin; JAK2, Janus kinase 2; LPS, lipopolysaccharide; STAT3, signal transducer and activator of transcription 3.

After IL-6R complex interacts with gp130, it can lead to the activation of receptor-bound JAK2 and following PI3K/protein kinase B (Akt) and STAT3 (53, 54). Given these, we examined the phosphorylation levels of JAK2, Akt, and STAT3. Western blot showed that the phosphorylation levels of JAK2 (Fig. 6*A*), Akt (Fig. 6*B*), and STAT3 (Fig. 6*C*) were all increased in the cerebral tissues of Fut8^+/−^ mice, compared with that in the Fut8^+/+^ mice under either normal condition or LPS treatment. Importantly, l-fucose administration significantly suppressed these increases in the phosphorylation levels (Fig. 6, *A*–*C*). The coimmunoprecipitation results revealed an elevated association between gp130 and IL-6R and a reduction in core fucosylation of gp130 in the Fut8^+/−^::*hIL6*-*Luc* mice when compared with the Fut8^+/+^::*hIL6*-*Luc* mice. Importantly, these effects were ameliorated by exogenous l-fucose (Fig. 6*D*). These results reveal that core fucosylation is closely involved in the pathogenesis of neuroinflammation, which may be mediated by the gp130–JAK2–Akt–STAT3 signaling pathway.

### Effects of l-fucose on neuroinflammation in Fut8^+/+^ mice

**Figure 7:**
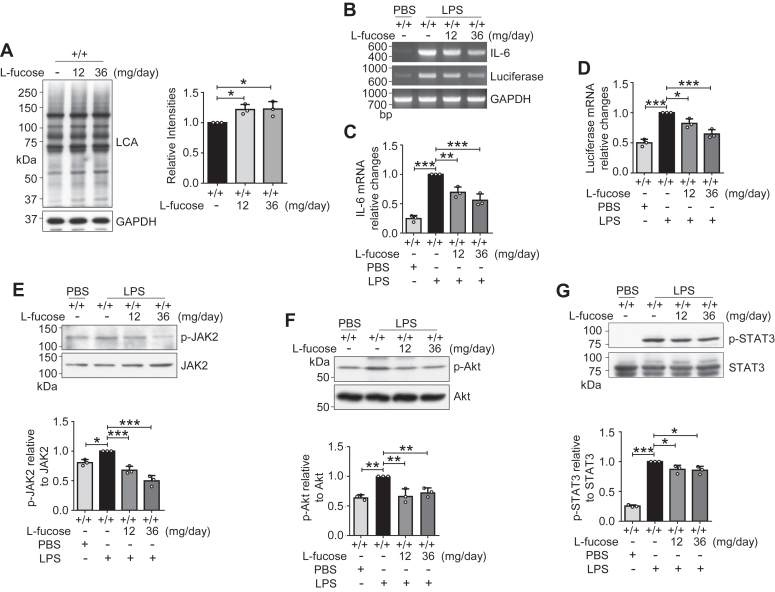
**Effects of****l****-fucose on fucosylation and signaling pathway in Fut8**^**+/+**^**mice.***A*, the brain tissues were obtained from the pretreated Fut8^+/+^ mice as described for Figure 1*B*. The fucosylation levels were detected by lectin blot, evidenced by LCA lectin. The quantitative data were analyzed from all the bands by one-way ANOVA test and shown as the mean ± SD from three independent experiments. The ratio of LCA *versus* GAPDH of Fut8^+/+^ mice treated without l-fucose was set as 1.0. ∗*p* < 0.05. The brain tissues were obtained from the pretreated Fut8^+/+^::*hIL6*-*Luc* mice as outlined for Figure 1*B*. Post 4 h after intraperitoneal injection, the mRNA levels of IL-6 and luciferase were detected by RT–PCR (*B*) and real-time PCR (*C* and *D*). The quantitative data were calculated by one-way ANOVA test and shown as the mean ± SD from three independent experiments. GAPDH was used as an internal control. The value of IL-6 or luciferase *versus* GAPDH of Fut8^+/+^::*hIL6*-*Luc* mice treated without l-fucose and injected with LPS was set as 1.0. ∗*p* < 0.05; ∗∗*p* < 0.01; ∗∗∗*p* < 0.001. *E*–*G*, Western blot examined the expression of p-JAK2 and JAK2 (*E*), p-Akt and Akt (*F*), and p-STAT3 and STAT3 (*G*). The ratios of p-JAK2 against JAK2, p-Akt against Akt, or p-STAT3 against STAT3 of Fut8^+/+^::*h*IL6-*Luc* mice treated without l-fucose and injected with LPS were set as 1.0. Data were shown as the mean ± SD from three independent experiments. ∗*p* < 0.05; ∗∗*p* < 0.01; ∗∗∗*p* < 0.001 (one-way ANOVA test). Akt, protein kinase B; *hIL6*, human IL-6 gene; IL, interleukin; JAK2, Janus kinase 2; LCA, *Lens culinaris* agglutinin; LPS, lipopolysaccharide; STAT3, signal transducer and activator of transcription 3.

As described previously, exogenous l-fucose exerted an antineuroinflammatory effect in the Fut8^+/−^ mice. Given this, we next asked whether l-fucose also exerts an inhibitory effect on the neuroinflammation in the Fut8^+/+^ mice. The LCA lectin blot result showed that the core fucosylation was increased after l-fucose administration, even in the Fut8^+/+^ mice (Fig. 7*A*). The RT–PCR and real-time PCR results showed that l-fucose treatment also alleviated the increased mRNA expression levels of IL-6 and luciferase in the Fut8^+/+^::*hIL6*-*Luc* mice stimulated with the LPS (Fig. 7, *B*–*D*). Consistent with the data obtained from the Fut8^+/−^::*hIL6*-*Lu*c mice, the phosphorylation levels of JAK2, Akt, and STAT3 upon LPS were significantly suppressed by the pretreatment with L-fucose (Fig. 7, *E*–*G*). These results strongly suggest that l-fucose generally exerts an antineuroinflammatory effect regardless of the Fut8 genotypes.

## Discussion

**Figure 8:**
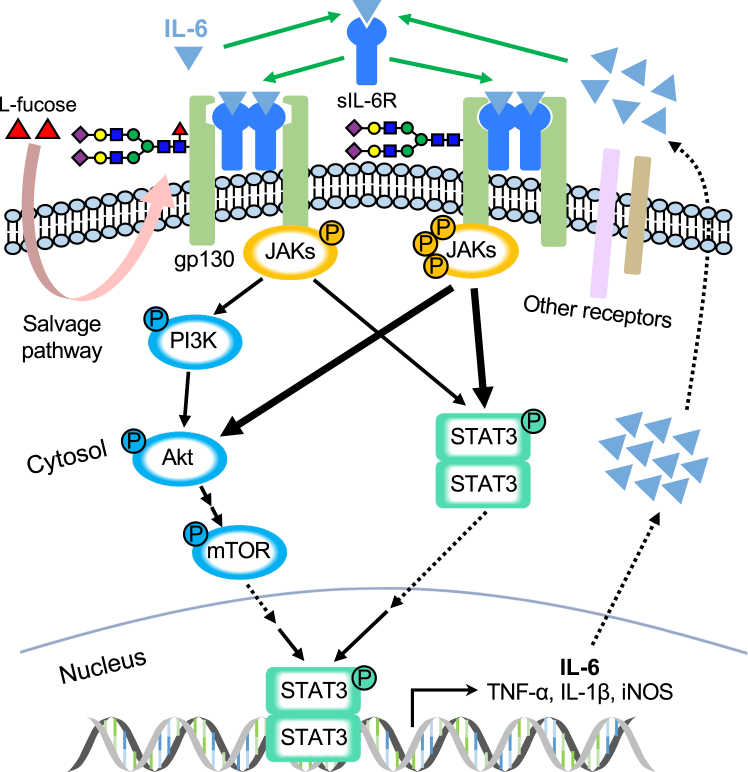
**Schematic diagram of the proposed molecular mechanism for neuroinflammation regulated by core fucosylation.** Based on our observations in the present study, core fucosylation could negatively regulate neuroinflammation induced by LPS, that is, lower core fucosylation as shown in Fut8^+/−^ mice enhanced expression of proinflammatory cytokines, such as IL-6, TNF-α, IL-1β, and iNOS, and microglial activation to induce neuroinflammation, which could be significantly suppressed by increasing core fucosylation using exogenous l-fucose. Considering IL-6 signaling is one of the main signaling pathways involved in neuroinflammation, and IL-6R can bind to the coreceptor gp130 to activate downstream JAK–STAT signaling pathway (27, 28), we believe that the core fucosylation on gp130 may give a significant impact both *in vitro* and *in vivo*, as observed in this study. The molecular mechanism can be postulated in that lack of core fucosylation of gp130 induces its property conformation for IL-6R binding. A similar phenomenon has been observed in the binding of IgG1 to FcγRIIIa, which was proved by structural biology studies (102, 103). Of course, we do not exclude other target glycoproteins besides gp130 since the core fucosylation is highly expressed in brain tissues and modifies many other cytokine receptors, such as TGF-β and TNF-α receptors, which may also positively and negatively regulate neuroinflammation. IL, interleukin; iNOS, inducible nitric oxide synthase; JAK, Janus kinase; LPS, lipopolysaccharide; STAT, signal transducer and activator of transcription; TGF-β, transforming growth factor-beta; TNF-α, tumor necrosis factor-alpha.

The present study demonstrated that l-fucose exerts therapeutic efficacy against LPS-induced neuroinflammation in the Fut8^+/−^ mice. We concluded that core fucosylation plays a critical role in antineuroinflammation, and the higher neuroinflammatory responses in the Fut8^+/−^ mice are attenuated by the administration of the exogenous l-fucose. As a plausible molecular mechanism, we speculate that the defective core fucosylation on some critical target proteins, such as gp130, results in its conformational changes, which accelerate IL-6R binding to the receptor, subsequently overactivating the downstream gp130–JAK2–Akt–STAT3 signaling pathway to further produce proinflammatory cytokines (Fig. 8).

*N*-glycosylation is one of the major post-translational modifications of proteins, which correlates to protein structure and function, including correct protein folding, stability, maturation, and protein–protein interaction (55, 56). Core fucosylation occurs exclusively on the core of *N*-glycans, which is catalyzed by Fut8 and is associated with numerous physiological and pathological processes (1, 57). For instance, the elevated level of core fucosylated α-fetoprotein is a reliable biomarker for hepatocellular carcinoma (58, 59). And increments in core fucosylation of serum proteins have been related to the increased risk of metastasis in prostate cancer, which can be a valuable biomarker for the detection of prostate cancer (60). Furthermore, lack of core fucosylation can enhance the affinity of human IgG1 binding to FcγRIIIa, which increases the antibody-dependent cellular cytotoxicity and improves the efficacy of anticancer chemotherapeutics both *in vivo* and *in vitro* (51, 61, 62). Consistently, the lack of core fucosylation in mouse IgG2 confers a 10-fold increased affinity for binding to FcγRIV (63). Thus, core fucosylation plays a vital role in numerous physiological and pathological processes, and its dysregulation can be associated with various diseases.

Core fucosylation exerts various biological functions that vary among diverse cell types. Fut8^−/−^ mice showed an emphysema-like pulmonary disorder because of an aberration in transforming growth factor beta 1 receptor activation and downstream signaling pathway (9). In addition, Fut8^+/−^ mice exhibited a more significant increase in sensitivity to a cigarette smoke–induced emphysema model than Fut8^+/+^ control mice (37). Consistent with these data, we found that the expression levels of IL-6 in lung tissues were significantly increased in Fut8^+/−^ mice relative to the Fut8^+/+^ mice. Furthermore, the treatment with exogenous l-fucose significantly suppressed the IL-6 expression (Fig. S5, *A*–*C*). Our findings, accompanied by the previous data, strongly suggest that core fucosylation negatively regulates inflammation. However, it is not always the case. We also noticed that the expression levels of IL-6 in spleen tissues were significantly decreased in Fut8^+/−^ mice compared with the Fut8^+/+^ mice, and the exogenous l-fucose significantly upregulated the IL-6 expression (Fig. S5, *D*–*F*). This observation can be supported by previous studies in which the deficiency of core fucosylation in CD14 could impair Toll-like receptor 4 signaling in mouse embryonic fibroblasts (64) and reduce the activation of RAW264.7 cells upon LPS stimulation (65). These outcomes suggest that the regulation of inflammation *via* core fucosylation may vary in different tissues. However, the precise mechanisms behind these tissue-specific variations require further investigation. We also speculate that the cause of altered inflammation may be related to gp130 expression level and function in different tissues. Several studies reported that gp130 is mainly involved in neuroinflammation and neurodegeneration in the brain (66, 67) and is crucial for regulating inflammation and tissue repair in the lung (68, 69). Nevertheless, it is also essential for developing, surviving, and activating immune cells in the spleen (70). It is worth noting that our previous study reported that lack of core fucose could induce a schizophrenia-like abnormal behavior (10, 32), which is possibly because of the neuroinflammation in the CNS and immune dysfunction (34, 35, 71). The deficiency of core fucosylation can upregulate the sensitivity of microglia and astrocytes to inflammatory stimuli and continuously regulate the neuroinflammation (36). Consistently, this study found that lower core fucosylation leads to higher sensitivity to inflammatory stimulators and induces severe neuroinflammatory status in Fut8^+/−^ mice, which exogenous l-fucose could attenuate. Thus, core fucosylation may play a critical role in regulating neuroinflammation in the brain.

Neuroinflammation refers to the innate immune response that takes place in the brain or spinal cord, which is associated with several neurodegenerative diseases, including Parkinson's disease, Alzheimer's disease, multiple sclerosis, major depressive disorder, and amyotrophic lateral sclerosis (42, 72). Microglia, the principal players in neuroinflammation, can secrete proinflammatory cytokines when activated, triggering immune responses and recruiting other immune cells to the site of injury or damage. It has been determined that overactivated microglia can generate a cohort of proinflammatory mediators, which may subsequently diminish neuronal plasticity, impair memory, and is generally considered a significant contributor to the development and progression of neurodegenerative disorders (44, 73). This study found that 2 weeks of l-fucose administration can significantly decrease the expression of proinflammatory cytokines and microglial activation triggered by LPS, which could be a new strategy for treating neurodegenerative disorders.

Furthermore, free l-fucose through the metabolism pathway of the GDP-fucose (Fig. 1*A*) can be utilized by fucosyltransferases, which plays a vital role in immune cell development, including macrophage polarization and function regulation (7). Our previous studies found that 2-fluoro-l-fucose, an inhibitor of fucosylation, could block cellular fucosylation in primary astrocytes (36) and two pancreatic adenocarcinoma cell lines, PANC-1 and MIA PaCa-2 cells in quite different doses (13), which indicates that the salvage pathway may differently affect on cellular fucosylation among cell lines or cell types. Interestingly, research by Freeze’s group reported that Fut8 could preferentially utilize the GDP-fucose originating from the exogenous fucose, whereas the GDP-fucose deriving from endogenous fucose was used by other fucosyltransferases to modify *N*-glycan antennae (40), which may partly explain our finding here to show that exogenous l-fucose could increase the core fucosylation downregulated in Fut8^+/−^ mice. Furthermore, these results are consistent with previous research, which demonstrated that oral l-fucose supplementation rescued the intracellular fucosylation. It remains unclear whether core fucosylation increased and markedly improved the neurological phenotype as well as the growth in one individual with biallelic GDP-l-fucose synthase variants (4). In addition, recently, it was reported that l-fucose was an effective agent for harmlessly enhancing intratumoral immune cells and immunotherapy efficacy in melanoma (74). Moreover, it is worth noting that l-fucose has been demonstrated as a generally safe and well-tolerated therapeutic agent in patients with leukocyte adhesion deficiency II, also known as SLC35C1-CDG (75, 76). Furthermore, fucoidan, a form of sulfated l-fucose polymers, has multiple biological and pharmacological activities, such as anticancer, antiproliferation, antioxidation, etc. (77, 78, 79). It has already been investigated for its potential use as a dietary supplement or synergistic anticancer agent in combination with chemotherapeutic drugs (80, 81, 82). However, the oral bioavailability of fucoidan could be low because of its highly polar nature and molecular weight (83, 84). Based on the observation that l-fucose enhanced the core fucosylation to exert an antineuroinflammatory effect both in Fut8^+/+^ and Fut8^+/−^ mice in the present study, we speculate that the effects of fucoidan on multiple cancers may be related to the anti-inflammatory effect of its monomer, l-fucose, to a certain extent.

It is well known that the neuroinflammatory responses mediated by microglia can be regulated by many signaling molecules in the proinflammatory signaling pathway, such as JAK2–STAT3 (85, 86). It can be activated by several cytokines and growth factors, such as IL-6 and erythropoietin (27, 87). In trans IL-6 signaling, IL-6 binds to the sIL-6R in circulation, then interacts with gp130, which has nine potential *N*-glycosylation sites (88), leading to the activation of JAK2–Akt–STAT3 signaling pathway and subsequent generation of proinflammatory cytokines and chemokines. *N*-glycosylation is crucial for gp130 stability, and the proteasomal degradation pathway can degrade unglycosylated gp130 before reaching the cell surface (89). The present study showed that core fucosylation of gp130 plays an essential role in the regulation of neuroinflammation *via* the gp130–JAK2–Akt–STAT3 signaling pathway (Fig. 8). Aberrant activation of the JAK2–Akt–STAT3 pathway can contribute to the development of various inflammatory diseases (90), which can be suppressed by the supplement of l-fucose (Fig. 6).

The core fucosylation is highly expressed in the brain tissues, as evidenced by MS analysis (Fig. 1). There are 79.5% of most major core fucosylated *N*-glycans that contain monofucose in Fut8^+/+^ mice whereas 57.9% in the Fut8^+/−^ mice. The treatment with l-fucose could upregulate the core fucosylation from 57.9% to 63.2% in the brain of Fut8^+/−^ mice (Fig. 1*D*). Although the increase in total core fucosylation by l-fucose was slight, its impact on neuroinflammation was significant. It could be explained that the exogenous l-fucose promotes core fucosylation on some important glycosylation sites and/or some target glycoproteins, such as gp130, as described previously, rather than total glycoproteins. We do not exclude other plausible mechanisms for the l-fucose effects. For instance, α1,3-fucosylation of low-density lipoprotein receptor–related protein 6 could promote its endocytosis, resulting in the inhibition of Wnt–β-catenin signaling, which can be reversed by the exogenous l-fucose (91, 92). Detailed information is required for further study. It is worth noting that the therapeutic impact of l-fucose on neuroinflammation was also observed in the Fut8^+/+^ mice (Fig. 7). Taken together, these results provide a notion that exogenous l-fucose exerts an anti-inflammatory efficacy *via* regulating core fucosylation. Therefore, we propose that l-fucose can be helpful as an essential supplementation.

The present study demonstrates that core fucosylation negatively modulates the severity of the LPS-induced neuroinflammation and that the exogenous l-fucose efficiently attenuates the neuroinflammation. Our findings may provide a novel concept of therapeutic l-fucose application in treating or preventing neurodegenerative diseases.

## Experimental procedures

### Antibodies and reagents

The experiments were performed using the following antibodies and reagents: biotinylated LCA (J207), which preferentially recognizes core fucose, was obtained from J-Oil Mills. The anti-Fut8 antibody (sc-271244) was obtained from Santa Cruz Biotechnology. The antibodies against GAPDH (G9545), α-Tubulin (T6199), the peroxidase-conjugated secondary antibody against mouse IgG (AP124P), and LPS purified from *Escherichia coli* O111:B4 (L2630) were from Sigma. The anti-Phospho-JAK2 (catalog no.: 3771), anti-JAK2 (catalog no.: 3230), anti-Phospho-Akt (catalog no.: 4060), anti-Akt (catalog no: 9272), anti-Phospho-STAT3 (catalog no.: 9145), anti-STAT3 (catalog no.: 9139) antibodies and the secondary antibody about horseradish peroxidase–conjugated goat against rabbit (catalog no.: 7074) were purchased from Cell Signaling Technology. The antibody against ionized calcium-binding adaptor molecule-1 (Iba-1) (catalog no.: 019-19741) was from Wako. The anti-gp130 (catalog no.: A304-929A), anti-IL-6R (catalog no.: MA5-29721), and antiluciferase (catalog no.: 35-6700) antibodies were from Thermo Fisher Scientific. Ab-Capcher MAG2 was purchased from ProteNova. ABC kit (catalog no.: PK-4000) was from Vector Laboratories. The goat anti-rabbit antibody Alexa Fluor 488 (catalog no.: A-11008) and goat antimouse antibody Alexa Fluor 647 (catalog no.: A-21236) were from Invitrogen. l-Fucose (F0065) was purchased from TCI.

### Animals

All animal experiments complied with protocols approved by the Animal Care and Use Committee of the Graduate School of Pharmaceutical Sciences, Tohoku Medical and Pharmaceutical University. Fut8^+/+^ littermates and Fut8^+/−^ mice were obtained by intercrossing the ICR genetic background heterozygous mice (10). Generation and analysis of the *hIL6*-BAC-*Luc* reporter transgenic mice were previously reported (21). The Fut8^+/−^ mice were mated with *hIL6*-BAC-*Luc* reporter transgenic mice to produce Fut8^+/+^::*hIL6*-*Luc* and Fut8^+/−^::*hIL6*-*Luc* compound transgenic mice. All experiments were conducted with 5- to 6-week-old male and female mice, male mice with a weight range of approximately 28 to 30 g and female mice with a weight range of approximately 26 to 28 g. Mice were housed in groups under standard vivarium conditions (12 h light–dark cycle, lights on from 7:00 to 19:00, 22 °C ± 2 deg. ambient temperature, and 55 ± 5% relative humidity) with free access to food and water. The mice were orally administrated l-fucose twice a day *via* oral gavage, with incremental doses of 4, 12, and 36 mg/day. The dosage of l-fucose was determined based on our previous study (93) and clinical research (4), where approximately 10 to 20 mg/day per 30 g body weight of l-fucose was found to be effective.

### Cell culture

The mouse microglia cell line BV-2 was kindly gifted by Professor Elisabetta Blasi (University of Modena and Reggio Emilia, Modena) and cultured in Dulbecco's modified Eagle's medium with 10% fetal bovine serum under a humidified atmosphere at 37 °C and 5% CO_2_.

### Establishment of Fut8-KO cells

The pSpCas9(BB)-2A-GFP (PX458) plasmid was obtained from Addgene (plasmid ID: 48138). BV-2 Fut8-KO cells were constructed *via* guide RNA (5′-ATTCGTCCACAACCTTGGC-3′) targeted to mouse Fut8 gene localized adjacent to Cas 9 in the pSpCas9(BB)-2A-GFP vector. The plasmid was electroporated into the BV-2 according to the manufacturer's instructions (Amaxa cell line Nucleofector kit V; Lonza). After 48 h post-transfection, GFP-positive cells were sorted using FACSAria II (BD Bioscience) and subsequently seeded into 96-well plates to establish single clones. Genomic DNA was amplified *via* PCR using the following primers: forward primer (5′-CCCCATTGTAGACAGCCTCC-3′); reverse primer (5′-ACAGTCTCTAGGGTCTGGAAC-3′), and sequenced with the reverse primer.

### HPLC separation of nucleotide sugars

Nucleotide sugars were purified from brain tissues using the protocol (94, 95). The column was Inertsil ODS-4, 4.6 × 250 mm, 3 μm particle size, and flow rate was set to 0.6 ml/min. Buffer A (200 mM triethylamine, adjusted pH to 6.0 with acetic acid) was used for equilibration, and buffer B (80% of A plus 20% acetonitrile) was used as an eluent. Elution gradient in the long column was conducted as follows: 100% buffer A for 35 min, 0% to 77% linear gradient of buffer B for 40 min, 77% to 100% buffer B for 1 min, 100% buffer B for 14 min, 100% buffer A for 1 min, and 100% buffer A for 20 min (95, 96). Retention time under separation conditions was approximately 24.54 min for GDP-fucose.

### LC–MS analysis of *N*-glycans from mice hippocampus

*N*-glycans from cell membrane proteins from each three mice hippocampus, of Fut8^+/+^ mice, Fut8^+/−^ mice, and Fut8^+/−^ mice treated with 36 mg/day l-fucose, were released with PNGaseF (97), labeled with aminoxyTMT6 reagent (Thermo Fisher Scientific) after the treatment of desialylation with acetic acid and then analyzed by LC–electrospray ionization MS, according to previous procedures (98).

### Imaging of luciferase activity *in vivo* and *ex vivo*

*In vivo*, bioluminescence imaging was conducted using an *IVIS* (IVIS Lumina Series III; PerkinElmer) as previously described (21). Briefly, the Fut8^+/+^::*hIL6*-*Luc* and Fut8^+/−^::*hIL6*-*Luc* transgenic mice were intraperitoneally injected with 75 mg/kg d-luciferin (Promega) 4 h after the intraperitoneal administration of PBS or LPS at a dose of 1 mg/kg. Subsequently, the anesthetized mice were placed in a light-sealed chamber, and the luciferase activity was imaged for 60 s to monitor the neuroinflammation. Regarding the *ex vivo* imaging, the brain tissues were isolated from the Fut8^+/+^::*hIL6*-*Luc* and Fut8^+/−^::*hIL6*-*Luc* transgenic mice euthanized immediately after the administration of d-luciferin, incubating the brain samples in 300 μg/ml d-luciferin in PBS. Luminescence emitted from the cerebral region of the mice was quantified with Living Image software (PerkinElmer).

### Immunoprecipitation

Brain tissues were rapidly extracted on ice, and each 50 mg tissue was homogenized in 500 μl radioimmunoprecipitation assay buffer (20 mM Tris–HCl, pH 7.5, 150 mM NaCl, 2 mM EDTA, 0.1% SDS, 1% NP-40, and 1% protease and phosphatase inhibitors) with φ2.0 Zirconia Beads by Micro Smash MS-100 (Digital Biology), based on the manufacturer’s instructions. BV-2 cells were washed with cold PBS for three times and then lysed with the cell lysate buffer (20 mM Tris–HCl, 150 mM NaCl, pH 7.4, and 1% Triton X-100) and 1% protease and phosphatase inhibitors. After centrifugation at 15,000 rpm for 15 min, the supernatants were collected, and the concentration was detected by the BCA protein assay kit (Pierce). For immunoprecipitation, 1.5 μl anti-gp130 antibody was combined with 15 μl Ab-Capcher MAG2 at 4 °C for 2 h with MT-360 Micro Tube Mixer. After washing the mixture three times, the same amounts of proteins (500 μg) from each tissue or cell were immunoprecipitated at 4 °C overnight. And then, these immunoprecipitates were washed twice with PBS and detected by lectin blot and Western blot.

### Western blot and lectin blot

Western blot and lectin blot were performed as follows: proteins (10 μg) or immunoprecipitants (10 μl) were equally loaded into 7.5% or 12% SDS-PAGE at 100 V and then transferred to polyvinylidene difluoride membranes (MilliporeSigma) at 10 V for 1 h. After blocking (5% bovine serum albumin for lectin blot/5% nonfat dry milk for Western blot) for 1 h at room temperature, the membranes were stained with LCA lectin or indicated primary antibodies at 4 °C overnight. After washing four times, the membranes were incubated with appropriate secondary antibodies. Based on the manufacturer's instructions, immunoreactive bands were detected using an immobilon Western Chemiluminescent Horseradish Peroxidase Substrate (Millipore).

### Immunofluorescence

After intraperitoneal injection for 4 h, the mice in different experimental groups were deeply anesthetized with pentobarbital sodium and perfused transcardially with 50 ml PBS, afterward intracardially perfused with 50 ml 4% paraformaldehyde in 0.01 M PBS, and then removed the brains to 4% paraformaldehyde for further postfixed, followed by dehydrating using 10, 20, and 30% sucrose, respectively. A cryostat sectioned the brains at 40 μm, and the frozen sections were collected in 24-well plates containing PBS. After being permeabilized with PBS containing 0.3% Triton X-100 for 30 min, the brain slices were incubated with PBS solution containing 3% bovine serum for another 30 min at room temperature, followed by further incubation with anti-Iba-1 (1:200 dilution) antibody overnight at a 4 °C room temperature. After that, the brain slices were washed three times with PBS every 10 min and then incubated with a secondary antibody (1:500 dilution) for 2 h at room temperature. Finally, the brain slices were marked by 4′,6-diamidino-2-phenylindole for 10 min in the dark, mounted on glass slides with 30% glycerin, and imaged using ZEISS LSM 900 confocal microscope. The number of Iba-1-labeled microglia was quantified using ImageJ software (NIH).

### RT–PCR and real-time PCR for mRNA expression analysis

RNAs were extracted with TRIzol reagent (Invitrogen), and 1 μg of total RNA was reverse-transcribed into complementary DNA by PrimeScript RT reagent with gDNA Eraser (Takara) according to the manufacturer's instructions. The sequences of primers for RT–PCR and real-time PCR are listed in Tables 1 and 2, respectively. The RT–PCR products were subjected to electrophoresis using 1.5% agarose gels containing ethidium bromide. Real-time PCR assays were executed using a TB Green Premix Ex Taq II (Tli RNaseH Plus) (Takara), and the conditions were as follows: initial denaturation at 95 °C for 30 s, then 40 cycles of denaturation at 95 °C for 5 s followed by annealing and extension at 60 °C for 30 s.

**Table 1:** Primer sequences for RT–PCR

Target gene	Primer sequences (5′-3′)	
	Forward sequences	Reverse sequences
mIL-6	AAGAGACTTCCATCCAGTTGGC	CACTCCTTCTGTGACTCCAGC
Luciferase	AGAACTGCCTGCGTGAGATT	GGTGTTGGAGCAAGATGGAT
TNF-α	CAGCCGATGGGTTGTACCTT	CCGGACTCCGCAAAGTCTAA
IL-1β	ATCTGGGATCCTCTCCAGCC	CTGGAAGGTCCACGGGAAAG
iNOS	ATGACTCCCAGCACAAAGGG	ACGCTGAGTACCTCATTGGC
Iba-1	TCTGAGGAGCTATGAGCC	TGCTGTCATTAGAAGGTCC
GAPDH	ACTCCACTCACGGCAAATTC	CCCTGTTGCTGTAGCCGTAT

**Table 2:** Primer sequences for real-time PCR

Target gene	Primer sequences (5′-3′)	
	Forward sequences	Reverse sequences
mIL-6	CTGCAAGAGACTTCCATCCAG	AGTGGTATAGACAGGTCTGTTGG
Luciferase	ACGATTTTGTGCCAGAGTCC	AGAATCTCACGCAGGCAGTT
TNF-α	AAGTCAACCTCCTCTCTGCC	CCGGACTCCGCAAAGTCTAA
IL-1β	TGTCTGAAGCAGCTATGGC	TGTTGATGTGCTGCTGCG
iNOS	ATGACTCCCAGCACAAAGGG	AACAGCACTCTCTTGCGGACC
Iba-1	TCTGAGGAGCTATGAGCC	TCCATGTACTTCGTCTTGAAGGC
GAPDH	GTCGTGGAGTCTACTGGTGTCTT	GAGATGATGACCCTTTTGGC

### 3D model building of *N*-glycosylated IL-6–sIL-6R–gp130 complex

Homology modeling of murine IL-6, sIL-6R, and gp130 was performed using SWISS-MODEL (99), and the template was the cryo-EM structure of human IL-6–sIL-6R–gp130 hexametric complex with stoichiometry of 2:2:2 (Protein Data Bank ID: 8D82) (52). Glycan Modeler tool in CHARMM-GUI (100) was used to build a 3D structural model of *N*-glycosylated murine IL-6–sIL-6R–gp130 complex. The homology model was used as a scaffold of the artificial *N*-glycosylation (GlcNAc2Man3GlcNAc2Fuc), and all the *N*-glycosylation sequons are utilized for modification (sIL6R: N32, N55, and N150; gp130: N43, N61, N83, N131, N157, N225, N388, N476, and N551). The glycan conformation was based on the CHARMM force field (101).

### Statistical analysis

All data are presented as the mean ± SD obtained from at least three independent experiments. Statistics were analyzed using a one-way ANOVA with Tukey's post hoc test or an unpaired Student's *t* test by GraphPad Prism 5.0 software (GraphPad Software, Inc). A probability value of *p* was considered as follows: n.s. (no significance), *p* > 0.05; ∗*p* < 0.05; ∗∗*p* < 0.01; and ∗∗∗*p* < 0.001.

## Data availability

All data are provided in the figures, tables, and supporting information in this article.

## Supporting information

This article contains supporting information.

## Conflict of interest

The authors declare that they have no conflicts of interest with the contents of this article.
