# Supplementary material for: Exogenous l-fucose attenuates neuroinflammation induced by lipopolysaccharide
Source: J Biol Chem. 2023 Dec 1;300(1):105513. doi: 10.1016/j.jbc.2023.105513 (PMC10772726; doi:10.1016/j.jbc.2023.105513)
Supplement: Supporting Figures S1–S5 [file mmc1.pdf]

Supplemental Fig. 1

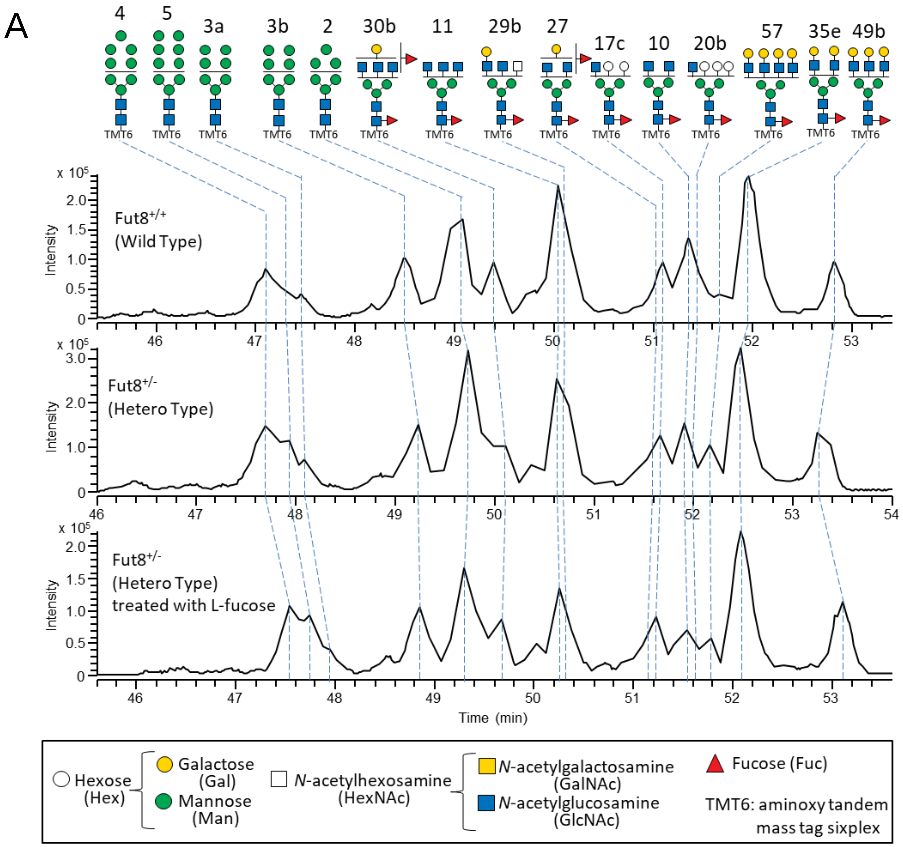

LC-MS analysis of *N*-glycans from the hippocampus of Fut8$^{+/+}$ mice, Fut8$^{+/-}$ and Fut8$^{+/-}$ mice treated with L-fucose. (A) The base peak chromatogram (BPC) for the LC-MS analysis of hippocampus *N*-glycans labeled with aminoxy tandem mass tag sixplex (TMT6) after treatment of desialylation. The deduced structures of the major *N*-glycans were shown. (B) Retention time, peak intensity, structure (component and No.), relative abundance, and diagnostic ion of core fucosylation and Lewis fucosylation for each *N*-glycan in LC-MS and MS/MS. (C-1) Extracted ion chromatograms (EICs) of major oligomannose type *N*-glycans (No. 2-5) and major complex type *N*-glycans (No. 28, 29, 30, 34 and 35) in MS, and EICs of diagnostic ions for core fucosylation and Lewis fucosylation in MS/MS. (C-2,3) MS/MS analysis of the representative non-fucosylated *N*-glycans (No. 28b and 34d), core fucosylated *N*-glycans (No. 29b and 35e) and core and Lewis fucosylated *N*-glycans (No. 30b and 36b). The diagnostic ions for core fucosylation, Lewis fucosylation, and Bisecting GlcNAc were indicated. (D-1,2,3) The intensities of major glycan species, including oligomannose *N*-glycans (No. 2-5), core fucosylated *N*-glycans (mono Fuc, No. 35b-e), core fucosylated *N*-glycans (mono Fuc, No. 10, 11, 14a, 17b,c, 20b, 21, 26c, 27, 29b,c, 30a,b, 32c, 33, 35b-e, 36a,b, 39b, 41a,c, 42a, 43a, 45b, 46b,c, 49a,b, 50a, 53a, 54a, 57, 58, based on diagnostic ion in MS/MS), core and Lewis fucosylated *N*-glycans (di Fuc, No. 30a,b) and core and Lewis fucosylated *N*-glycans (di Fuc, No. 18, 21, 27, 30a,b, 36b, 42a, 43a, 54a, 58, based on diagnostic ion in MS/MS) were shown here. Relative abundances (%) were calculated by setting the total intensities of major oligomannose *N*-glycans as 100%. Each dataset represented a mixture of 3 mice.

Supplemental Fig. 1

B

					Desialylated N-glycans labeled with aminoxyTMT6									Diagnostic ion in MS/MS	Diagnostic ion in MS/MS
					TMT6_129 22111506	TMT6_130 22111507	TMT6_131 22111508	TMT6_129 22111506	TMT6_130 22111507	TMT6_131 22111508	TMT6_129 22111506	TMT6_130 22111507	TMT6_131 22111508		
Theoretical mass (6ppm)			Structures		Retention time (min)			Intensity (EIC of theor.mass w 6ppm)			%			Core Fuc	Lewis Fuc
[M+H]+	[M+2H]2+	[M+3H]3+	Component	No.	F8W_Hip	F8H_Hip	F8Hf_Hip	F8W_Hip	F8H_Hip	F8Hf_Hip	F8W_Hip	F8H_Hip	F8Hf_Hip	669.388	512.198
1536.672	768.840	512.896	(Hex)2 + (Man)3(GlcNAc)2	1	52.92	53.38	53.16	22,800	34,000	15,700	0.89	0.87	0.60		
1698.725	849.867	566.914	(Hex)3 + (Man)3(GlcNAc)2	2	49.09	49.74	49.29	167,000	319,000	168,000	6.49	8.18	6.40		
1860.778	930.893	620.931	(Hex)4 + (Man)3(GlcNAc)2	3a	47.45	48.09	47.87	40,900	73,200	47,100	1.59	1.88	1.79		
				3b	48.48	49.24	48.85	102,000	154,000	108,000	3.96	3.95	4.11		
2022.83	1011.919	674.949	(Hex)5 + (Man)3(GlcNAc)2	4	47.09	47.69	47.54	83,800	149,000	109,000	3.26	3.82	4.15		
2184.883	1092.946	728.966	(Hex)6 + (Man)3(GlcNAc)2	5	47.26	47.94	47.75	50,800	115,000	94,700	1.97	2.95	3.61		
2346.937	1173.973	782.984	(Hex)7 + (Man)3(GlcNAc)2	6	47.68	48.27	48.01	2,620	9,580	4,880	0.10	0.25	0.19		
1618.727	809.867	540.248	(HexNAc)2 + (Man)3(GlcNAc)2	7	49.18	49.87	49.42	17,100	18,500	8,460	0.66	0.47	0.32		
1821.804	911.406	607.940	(HexNAc)3 + (Man)3(GlcNAc)2	8	47.35	47.84	47.75	23,700	24,600	10,000	0.92	0.63	0.38		
2024.886	1012.94695	675.634	(HexNAc)4 + (Man)3(GlcNAc)2	9	46.36	46.74	46.84	639	1,110	1,140	0.02	0.03	0.04		
1764.783	882.896	588.933	(HexNAc)2 (Deoxyhexose)1 + (Man)3(GlcNAc)2	10	51.35	51.92	51.54	137,000	156,000	71,000	5.32	4.00	2.70	+++	
1967.862	984.435	656.626	(HexNAc)3 (Deoxyhexose)1 + (Man)3(GlcNAc)2	11	50.04	50.63	50.25	224,000	254,000	136,000	8.70	6.51	5.18	+++	
2170.944	1085.976	724.320	(HexNAc)4 (Deoxyhexose)1 + (Man)3(GlcNAc)2	12a	49.09	49.74	49.42	3,380	4,720	3,470	0.13	0.12	0.13		
				12b	52.27	52.83	52.51	2,020	5,850	2,110	0.08	0.15	0.08		
1577.7006	789.354	526.572	(Hex)1 (HexNAc)1 + (Man)3(GlcNAc)2	13	–	–	–	0	0	0	0.00	0.00	0.00		
1723.756	862.382	575.257	(Hex)1 (HexNAc)1 (Deoxyhexose)1 + (Man)3(GlcNAc)2	14a	50.44	51.10	50.64	9,860	14,200	6,240	0.38	0.36	0.24	+	
				14b	51.99	52.48	52.16	9,630	10,100	6,910	0.37	0.26	0.26		
				14c	55.96	56.35	56.40	6,070	11,400	4,290	0.24	0.29	0.16		
1869.814	935.411	623.943	(Hex)1 (HexNAc)1 (Deoxyhexose)2 + (Man)3(GlcNAc)2	15a	54.22	54.72	54.77	2,310	3,760	2,900	0.09	0.10	0.11		
				15b	54.98	55.42	55.42	8,630	15,600	9,810	0.34	0.40	0.37		
1739.752	870.38	580.589	(Hex)2 (HexNAc)1 + (Man)3(GlcNAc)2	16a	47.53	48.09	47.95	9,420	13,500	10,200	0.37	0.35	0.39		
				16b	48.96	49.62	49.29	14,400	20,700	12,800	0.56	0.53	0.49		
1885.81	943.409	629.275	(Hex)2 (HexNAc)1 (Deoxyhexose)1 + (Man)3(GlcNAc)2	17a	47.66	48.33	48.12	3,280	6,430	3,380	0.13	0.16	0.13		
				17b	50.79	50.63	50.25	12,500	22,500	16,300	0.49	0.58	0.62	+	
				17c	51.10	51.67	51.22	96,600	129,000	93,200	3.75	3.31	3.55	+++	
2031.867	1016.4375	677.961	(Hex)2 (HexNAc)1 (Deoxyhexose)2 + (Man)3(GlcNAc)2	18	50.17	50.75	50.38	22,200	30,200	30,700	0.86	0.77	1.17		++
1901.804	951.406	634.607	(Hex)3 (HexNAc)1 + (Man)3(GlcNAc)2	19	49.40	50.12	49.67	28,100	55,500	30,800	1.09	1.42	1.17		
2047.862	1024.435	683.293	(Hex)3 (HexNAc)1 (Deoxyhexose)1 + (Man)3(GlcNAc)2	20a	48.30	48.98	48.65	6,520	12,500	9,970	0.25	0.32	0.38		
				20b	51.37	51.92	51.54	54,800	87,000	67,100	2.13	2.23	2.56	+++	
2193.92	1097.464	731.979	(Hex)3 (HexNAc)1 (Deoxyhexose)2 + (Man)3(GlcNAc)2	21	50.59	51.19	50.71	16,200	24,600	22,600	0.63	0.63	0.86	+	+
2063.8596	1032.434	688.625	(Hex)4 (HexNAc)1 + (Man)3(GlcNAc)2	22	–	–	–	0	0	0	0.00	0.00	0.00		
2209.918	1105.463	737.311	(Hex)4 (HexNAc)1 (Deoxyhexose)1 + (Man)3(GlcNAc)2	23	–	–	–	0	0	0	0.00	0.00	0.00		
2355.976	1178.492	785.997	(Hex)4 (HexNAc)1 (Deoxyhexose)2 + (Man)3(GlcNAc)2	24	–	–	–	0	0	0	0.00	0.00	0.00		
1780.778	890.893	594.265	(Hex)1 (HexNAc)2 + (Man)3(GlcNAc)2	25a	43.10	43.53	43.69	4,180	4,210	2,250	0.16	0.11	0.09		
				25b	45.41	45.92	45.93	3,970	3,750	2,280	0.15	0.10	0.09		
				25c	46.55	46.99	46.99	3,660	4,800	3,140	0.14	0.12	0.12		
				25d	49.81	50.63	50.25	4,600	8,870	3,170	0.18	0.23	0.12		
1926.836	963.922	642.951	(Hex)1 (HexNAc)2 (Deoxyhexose)1 + (Man)3(GlcNAc)2	26a	48.67	49.36	49.07	7,960	9,140	6,940	0.31	0.23	0.26		
				26b	49.27	50.00	49.55	13,000	19,600	10,200	0.51	0.50	0.39		
				26c	51.89	52.42	51.97	48,100	57,300	35,900	1.87	1.47	1.37	+++	
2072.894	1036.951	691.637	(Hex)1 (HexNAc)2 (Deoxyhexose)2 + (Man)3(GlcNAc)2	27	51.00	51.54	51.10	48,000	74,400	49,700	1.86	1.91	1.89	+	+++
1983.8596	992.434	661.958	(Hex)1 (HexNAc)3 + (Man)3(GlcNAc)2	28a	42.73	43.10	43.28	5,610	4,740	2,710	0.22	0.12	0.10		
				28b	47.45	47.94	47.87	8,980	12,000	6,550	0.35	0.31	0.25		
				28c	48.20	48.87	48.14	1,540	3,570	1,980	0.06	0.09	0.08		
2129.916	1065.462	710.644	(Hex)1 (HexNAc)3 (Deoxyhexose)1 + (Man)3(GlcNAc)2	29a	46.51	47.01	46.95	11,900	18,500	13,100	0.46	0.47	0.50		
				29b	50.04	50.63	50.25	70,100	97,700	72,700	2.72	2.51	2.77	+++	
				29c	50.63	51.10	50.70	14,900	22,200	16,200	0.58	0.57	0.62	++	
2275.973	1138.491	759.330	(Hex)1 (HexNAc)3 (Deoxyhexose)2 + (Man)3(GlcNAc)2	30a	48.96	49.62	49.29	27,800	40,000	24,100	1.08	1.03	0.92	+	++
				30b	49.40	50.00	49.67	81,100	104,000	87,800	3.15	2.67	3.34	++	++
2186.9391	1093.973	729.652	(Hex)1 (HexNAc)4 + (Man)3(GlcNAc)2	31	46.42	46.96	46.83	1,170	2,130	1,530	0.05	0.05	0.06		
2332.995	1167.0015	778.337	(Hex)1 (HexNAc)4 (Deoxyhexose)1 + (Man)3(GlcNAc)2	32a	45.55	46.06	46.00	1,100	1,660	1,340	0.04	0.04	0.05		
				32b	49.09	49.87	49.42	7,040	8,440	6,350	0.27	0.22	0.24		
				32c	49.92	50.63	50.12	20,700	39,600	21,800	0.80	1.02	0.83	+++	
2479.055	1240.032	827.024	(Hex)1 (HexNAc)4 (Deoxyhexose)2 + (Man)3(GlcNAc)2	33	48.30	48.98	48.72	10,200	18,600	11,000	0.40	0.48	0.42	+	
1942.831	971.920	648.282	(Hex)2 (HexNAc)2 + (Man)3(GlcNAc)2	34a	45.98	46.36	46.48	15,900	26,900	15,300	0.62	0.69	0.58		
				34b	47.11	47.69	47.54	11,700	16,700	10,200	0.45	0.43	0.39		
				34c	49.27	49.74	49.29	9,320	1,740	1,330	0.36	0.04	0.05		
				34d	50.30	50.88	50.38	33,700	40,000	17,700	1.31	1.03	0.67		
2088.89	1044.949	696.969	(Hex)2 (HexNAc)2 (Deoxyhexose)1 + (Man)3(GlcNAc)2	35a	46.26	46.76	46.79	7,210	14,500	9,630	0.28	0.37	0.37		
				35b	48.65	49.36	49.07	21,000	31,700	18,400	0.82	0.81	0.70	++	
				35c	49.81	50.37	50.03	48,100	60,900	50,600	1.87	1.56	1.93	+++	
				35d	51.55	49.74	51.66	43,300	53,700	40,800	1.68	1.38	1.55	+++	
				35e	51.96	52.48	52.09	241,000	323,000	223,000	9.36	8.28	8.50	+++	
2234.946	1117.977	745.654	(Hex)2 (HexNAc)2 (Deoxyhexose)2 + (Man)3(GlcNAc)2	36a	49.09	49.74	49.42	36,500	53,500	47,300	1.42	1.37	1.80	+++	
				36b	51.33	51.79	51.41	29,700	47,000	39,600	1.15	1.21	1.51	++	+
2104.8861	1052.947	702.301	(Hex)3 (HexNAc)2 + (Man)3(GlcNAc)2	37a	45.64	46.25	46.10	10,300	10,200	8,790	0.40	0.26	0.33		
				37b	46.81	47.27	47.26	10,200	18,200	10,700	0.40	0.47	0.41		
				37c	47.41	48.09	47.25	6,170	5,600	4,470	0.24	0.14	0.17		
2250.942	1125.975	750.986	(Hex)3 (HexNAc)2 (Deoxyhexose)1 + (Man)3(GlcNAc)2	38a	45.80	46.31	46.28	7,150	11,500	8,370	0.28	0.29	0.32		
				38b	48.65	49.49	49.07	3,470	5,430	6,430	0.13	0.14	0.24		
				38c	49.40	50.00	49.55	11,900	19,900	15,700	0.46	0.51	0.60		
				38d	51.91	52.42	51.97	7,230	11,700	8,490	0.28	0.30	0.32		
				38e	52.24	52.70	52.35	2,870	3,240	3,100	0.11	0.08	0.12		
2397.000	1199.004	799.672	(Hex)3 (HexNAc)2 (Deoxyhexose)2 + (Man)3(GlcNAc)2	39a	47.61	48.18	48.01	4,230	6,030	5,880	0.16	0.15	0.22		
				39b	48.46	49.24	48.84	16,400	23,100	21,000	0.64	0.59	0.80	+++	
2145.913	1073.4602	715.976	(Hex)2 (HexNAc)3 + (Man)3(GlcNAc)2	40a	47.46	48.03	47.75	2,030	2,630	2,190	0.08	0.07	0.08		
				40b	48.85	49.49	49.07	786	1,450	1,430	0.03	0.04	0.05		
				40c	50.95	51.54	51.10	1,150	2,450	1,360	0.04	0.06	0.05		
2291.968	1146.488	764.661	(Hex)2 (HexNAc)3 (Deoxyhexose)1 + (Man)3(GlcNAc)2	41a	50.04	50.63	50.12	11,700	25,600	14,000	0.45	0.66	0.53	++	
				41b	50.74	51.29	50.88	5,970	8,970	6,890	0.23	0.23	0.26		
				41c	52.72	53.19	52.95	17,100	22,100	15,400	0.66	0.57	0.59	++	
2438.026	1219.517	813.347	(Hex)2 (HexNAc)3 (Deoxyhexose)2 + (Man)3(GlcNAc)2	42a	49.27	50.00	49.55	18,400	33,100	26,400	0.71	0.85	1.01	+++	++
				42b	49.92	50.63	50.25	6,920	14,600	13,000	0.27	0.37	0.50		
				42c	52.25	52.30	52.42	3,260	3,250	4,920	0.13	0.08	0.19		
2584.084	1292.546	862.033	(Hex)2 (HexNAc)3 (Deoxyhexose)3 + (Man)3(GlcNAc)2	43a	48.15	48.86	48.54	21,400	39,500	30,200	0.83	1.01	1.15	+	+
				43b	49.09	49.87	49.42	1,750	3,530	2,320	0.07	0.09	0.09		
				43c	49.53	50.25	49.81	2,130	4,380	3,410	0.08	0.11	0.13		
2348.9921	1175.000	783.669	(Hex)2 (HexNAc)4 + (Man)3(GlcNAc)2	44	46.92	47.41	46.86	743	1,620	1,030	0.03	0.04	0.04		
2495.047	1248.0275	832.354	(Hex)2 (HexNAc)4 (Deoxyhexose)1 + (Man)3(GlcNAc)2	45a	48.87	49.62	49.29	6,570	10,900	9,580	0.26	0.28	0.36		
				45b	49.83	50.50	50.03	14,100	27,800	19,100	0.55	0.71	0.73	+++	
				45c	50.17	50.75	50.38	5,750	14,900	10,800	0.22	0.38	0.41		
				45d	51.67	52.04	51.66	4,080	6,450	4,330	0.16	0.17	0.16		
2641.106	1321.057	881.041	(Hex)2 (HexNAc)4 (Deoxyhexose)2 + (Man)3(GlcNAc)2	46a	47.96	48.73	48.37	4,200	6,650	4,620	0.16	0.17	0.18		
				46b	48.79	49.49	49.07	9,910	14,500	13,200	0.38	0.37	0.50	++	
				46c	49.40	50.12	49.55	6,160	14,800	12,400	0.24	0.38	0.47	+	
				46d	50.18	51.67	51.22	1,220	2,060	1,060	0.05	0.05	0.04		
2787.163	1394.0855	929.726	(Hex)2 (HexNAc)4 (Deoxyhexose)3 + (Man)3(GlcNAc)2	47a	47.26	47.84	47.69	1,900	3,320	2,190	0.07	0.09	0.08		
				47b	47.82	48.50	48.17	2,880	7,340	2,710	0.11	0.19	0.10		
2307.966	1154.4867	769.994	(Hex)3 (HexNAc)3 + (Man)3(GlcNAc)2	48a	–	–	–	0	0	0	0.00	0.00	0.00		
				48b	50.17	50.75	49.91	953	1,970	1,380	0.04	0.05	0.05		
				48c	51.19	51.79	51.35	3,720	7,980	5,700	0.14	0.20	0.22		
2454.021	1227.515	818.679	(Hex)3 (HexNAc)3 (Deoxyhexose)1 + (Man)3(GlcNAc)2	49a	50.89	51.41	51.00	18,400	41,800	23,100	0.71	1.07	0.88	+++	
				49b	52.83	53.25	53.11	91,900	125,000	101,000	3.57	3.21	3.85	+++	
2600.080	1300.544	867.365	(Hex)3 (HexNAc)3 (Deoxyhexose)2 + (Man)3(GlcNAc)2	50a	50.30	50.88	50.50	10,500	26,300	17,600	0.41	0.67	0.67	+	
				50b	52.25	52.70	52.33	14,100	29,700	21,600	0.55	0.76	0.82		
2746.137	1373.5725	916.051	(Hex)3 (HexNAc)3 (Deoxyhexose)3 + (Man)3(GlcNAc)2	51a	49.81	50.50	50.03	6,190	17,800	12,300	0.24	0.46	0.47		
				51b	51.79	52.30	51.88	3,450	7,030	5,030	0.13	0.18	0.19		
2511.045	1256.02645	837.687	(Hex)3 (HexNAc)4 + (Man)3(GlcNAc)2	52a	50.43	50.63	50.12	651	1,320	911	0.03	0.03	0.03		
				52b	–	50.88	50.80	0	1,050	725	0.00	0.03	0.03		
2657.100	1329.054	886.372	(Hex)3 (HexNAc)4 (Deoxyhexose)1 + (Man)3(GlcNAc)2	53a	50.04	50.63	50.12	9,880	20,000	14,500	0.38	0.51	0.55	++	
				53b	51.67	52.17	51.66	10,800	18,800	12,800	0.42	0.48	0.49		
2803.161	1402.08445	935.059	(Hex)3 (HexNAc)4 (Deoxyhexose)2 + (Man)3(GlcNAc)2	54a	49.18	49.87	49.42	11,700	20,900	18,600	0.45	0.54	0.71	++	+
				54b	51.10	51.67	51.22	5,700	6,400	3,680	0.22	0.16	0.14		
2949.219	1475.11345	983.745	(Hex)3 (HexNAc)4 (Deoxyhexose)3 + (Man)3(GlcNAc)2	55a	47.92	48.67	48.37	6,340	15,300	11,000	0.25	0.39	0.42		
				55b	50.50	51.10	50.64	3,740	6,400	3,680	0.15	0.16	0.14		
2673.098	1337.05295	891.705	(Hex)4 (HexNAc)4 + (Man)3(GlcNAc)2	56a	50.17	50.75	50.12	842	3,600	2,310	0.03	0.09	0.09		
				56b	50.74	51.10	50.64	1,240	4,580	3,700	0.05	0.12	0.14		
2819.153	1410.081	940.390	(Hex)4 (HexNAc)4 (Deoxyhexose)1 + (Man)3(GlcNAc)2	57	51.67	52.17	51.79	36,300	78,200	54,400	1.41	2.01	2.07	+++	
2965.211	1483.1095	989.076	(Hex)4 (HexNAc)4 (Deoxyhexose)2 + (Man)3(GlcNAc)2	58	51.35	51.79	51.35	9,870	27,300	18,500	0.38	0.70	0.70	+	+
3111.272	1556.13995	1037.763	(Hex)4 (HexNAc)4 (Deoxyhexose)3 + (Man)3(GlcNAc)2	59	50.79	51.41	50.88	4,720	14,800	10,500	0.18	0.38	0.40		
2876.178	1438.5927	959.398	(Hex)4 (HexNAc)5 + (Man)3(GlcNAc)2	60	–	–	–	0	0	0	0.00	0.00	0.00		
3022.236	1511.6217	1008.084	(Hex)4 (HexNAc)5 (Deoxyhexose)1 + (Man)3(GlcNAc)2	61	48.63	49.49	48.94	4,560	6,490	6,450	0.18	0.17	0.25		
3168.290	1584.649	1056.769	(Hex)4 (HexNAc)5 (Deoxyhexose)2 + (Man)3(GlcNAc)2	62a	47.63	48.33	48.01	2,640	6,190	2,900	0.10	0.16	0.11		
				62b	48.24	48.98	48.54	2,250	2,630	3,430	0.09	0.07	0.13		
3314.3516	1657.6797	1105.456	(Hex)4 (HexNAc)5 (Deoxyhexose)3 + (Man)3(GlcNAc)2	63a	47.41	47.69	47.46	1,080	2,060	2,600	0.04	0.05	0.10		
				63b	–	–	–	0	0	0	0.00	0.00	0.00		
1778.682	889.845	593.566	(Hex)3 (Sulph)1 + (Man)3(GlcNAc)2	64	–	–	–	0	0	0	0.00	0.00	0.00		
2168.846	1084.927	723.621	(Hex)2 (HexNAc)2 (Deoxyhexose)1 (Sulph)1 + (Man)3(GlcNAc)2	65	54.46	55.02	55.03	11,300	19,200	9,740	0.44	0.49	0.37		
							SUM	2,574,174	3,900,000	2,624,966	100	100	100		

Supplemental Fig. 1

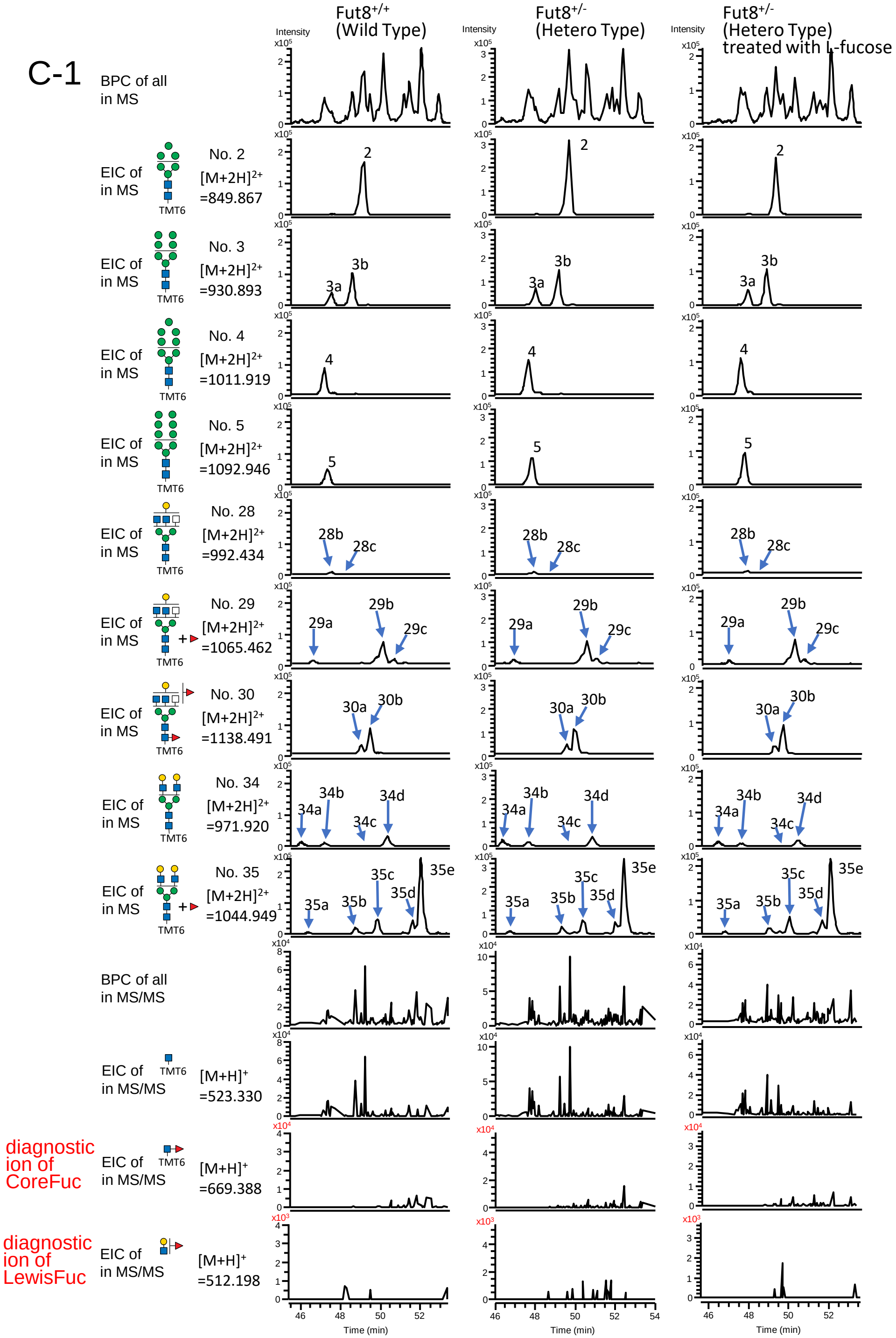

Supplemental Fig. 1

C-2

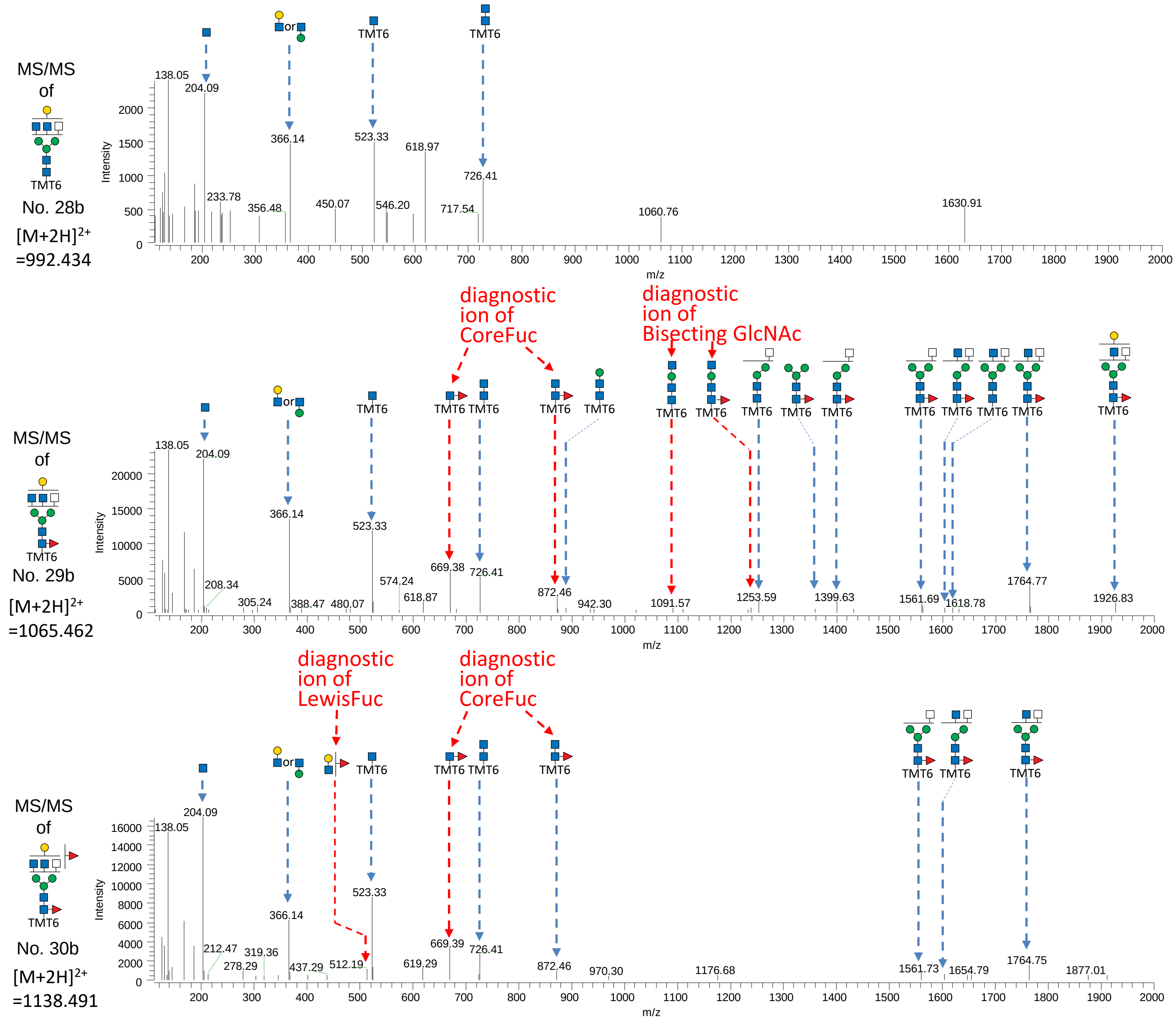

Supplemental Fig. 1

C-3

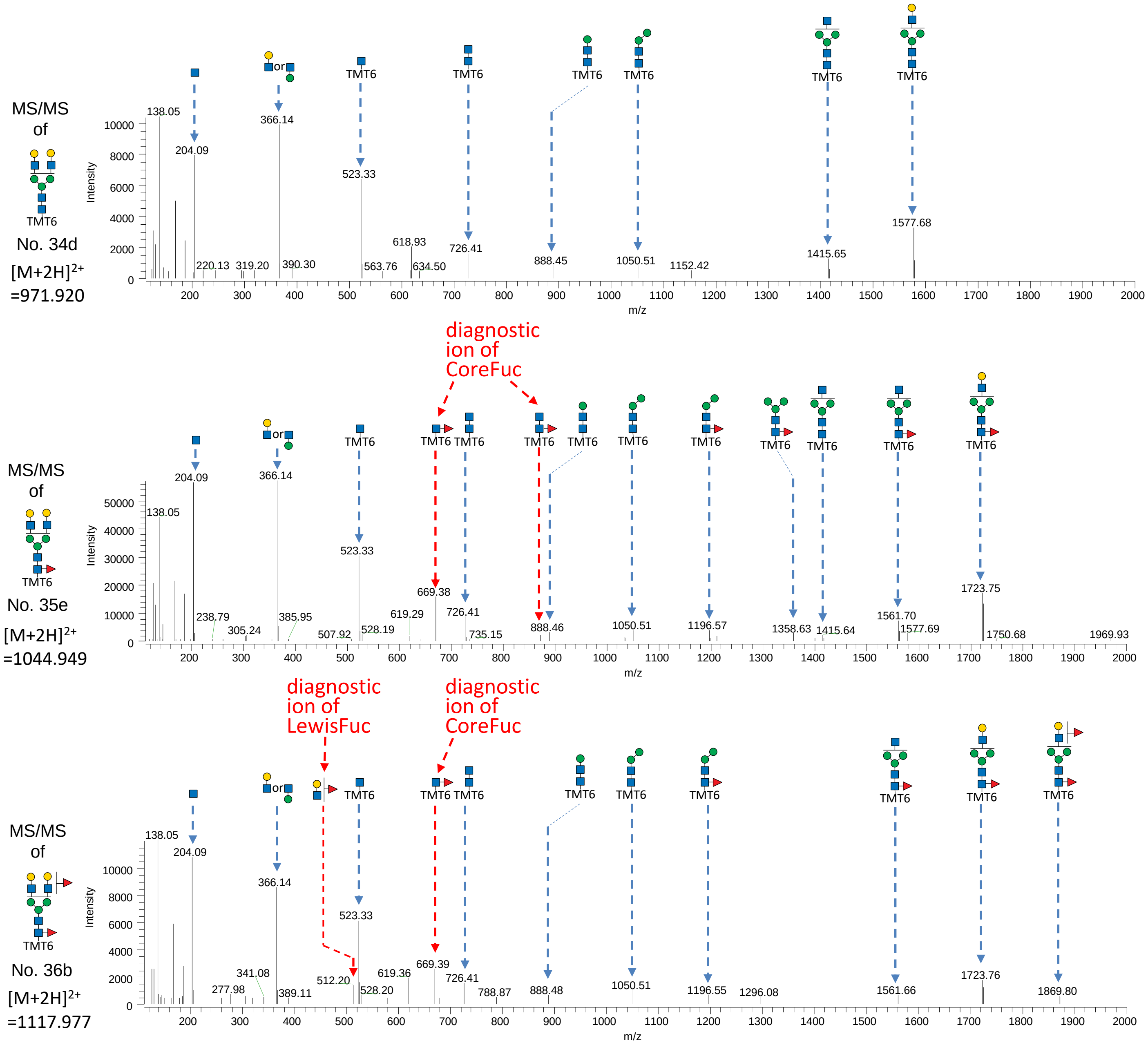

Supplemental Fig. 1

D-1

Fuc	*N*-glycans	No.	Intensity		
			Fut8+/+	Fut8+/−	Fut8+/− treated with L−fucose
−	total major oligomannose *N*-glycans	2	167,000	319,000	168,000
		3a	40,900	73,200	47,100
		3b	102,000	154,000	108,000
		4	83,800	149,000	109,000
		5	50,800	115,000	94,700
CoreFuc	most major core fucosylated *N*-glycans (mono Fuc)	35b	21,000	31,700	18,400
		35c	48,100	60,900	50,600
		35d	43,300	53,700	40,800
		35e	241,000	323,000	223,000
	total major core fucosylated *N*-glycans (mono Fuc, based on diagnostic ion in MS/MS)	10	137,000	156,000	71,000
		11	224,000	254,000	136,000
		14a	9,860	14,200	6,240
		17b	12,500	22,500	16,300
		17c	96,600	129,000	93,200
		20b	54,800	87,000	67,100
		21	16,200	24,600	22,600
		26c	48,100	57,300	35,900
		27	48,000	74,400	49,700
		29b	70,100	97,700	72,700
		29c	14,900	22,200	16,200
		30a	27,800	40,000	24,100
		30b	81,100	104,000	87,800
		32c	20,700	39,600	21,800
		33	10,200	18,600	11,000
		35b	21,000	31,700	18,400
		35c	48,100	60,900	50,600
		35d	43,300	53,700	40,800
		35e	241,000	323,000	223,000
		36a	36,500	53,500	47,300
		36b	29,700	47,000	39,600
		39b	16,400	23,100	21,000
		41a	11,700	25,600	14,000
		41c	17,100	22,100	15,400
		42a	18,400	33,100	26,400
		43a	21,400	39,500	30,200
		45b	14,100	27,800	19,100
		46b	9,910	14,500	13,200
		46c	6,160	14,800	12,400
		49a	18,400	41,800	23,100
		49b	91,900	125,000	101,000
		50a	10,500	26,300	17,600
		53a	9,880	20,000	14,500
		54a	11,700	20,900	18,600
		57	36,300	78,200	54,400
		58	9,870	27,300	18,500
CoreFuc + LewisFuc	most major core and Lewis fucosylated *N*-glycans (di Fuc)	30a	27,800	40,000	24,100
		30b	81,100	104,000	87,800
	total major core and Lewis fucosylated *N*-glycans (di Fuc, based on diagnostic ion in MS/MS)	18	22,200	30,200	30,700
		21	16,200	24,600	22,600
		27	48,000	74,400	49,700
		30a	27,800	40,000	24,100
		30b	81,100	104,000	87,800
		36b	29,700	47,000	39,600
		42a	18,400	33,100	26,400
		43a	21,400	39,500	30,200
		54a	11,700	20,900	18,600
		58	9,870	27,300	18,500

Supplemental Fig. 1

D-2

Fuc	*N*-glycans	No.	The relative abundances (%) were calculated by setting the total intensities of major oligomannose *N*-glycans as 100%.		
			Fut8+/+	Fut8+/−	Fut8+/− treated with L−fucose
−	total major oligomannose *N*-glycans	2	100	100	100
		3a			
		3b			
		4			
		5			
CoreFuc	most major core fucosylated *N*-glycans (mono Fuc)	35b	4.7	3.9	3.5
		35c	10.8	7.5	9.6
		35d	9.7	6.6	7.7
		35e	54.2	39.9	42.3
	total major core fucosylated *N*-glycans (mono Fuc, based on diagnostic ion in MS/MS)	10	30.8	19.3	13.5
		11	50.4	31.4	25.8
		14a	2.2	1.8	1.2
		17b	2.8	2.8	3.1
		17c	21.7	15.9	17.7
		20b	12.3	10.7	12.7
		21	3.6	3.0	4.3
		26c	10.8	7.1	6.8
		27	10.8	9.2	9.4
		29b	15.8	12.1	13.8
		29c	3.4	2.7	3.1
		30a	6.3	4.9	4.6
		30b	18.2	12.8	16.7
		32c	4.7	4.9	4.1
		33	2.3	2.3	2.1
		35b	4.7	3.9	3.5
		35c	10.8	7.5	9.6
		35d	9.7	6.6	7.7
		35e	54.2	39.9	42.3
		36a	8.2	6.6	9.0
		36b	6.7	5.8	7.5
		39b	3.7	2.9	4.0
		41a	2.6	3.2	2.7
		41c	3.8	2.7	2.9
		42a	4.1	4.1	5.0
		43a	4.8	4.9	5.7
		45b	3.2	3.4	3.6
		46b	2.2	1.8	2.5
		46c	1.4	1.8	2.4
		49a	4.1	5.2	4.4
		49b	20.7	15.4	19.2
		50a	2.4	3.2	3.3
		53a	2.2	2.5	2.8
		54a	2.6	2.6	3.5
		57	8.2	9.7	10.3
		58	2.2	3.4	3.5
CoreFuc + LewisFuc	most major core and Lewis fucosylated *N*-glycans (di Fuc)	30a	6.3	4.9	4.6
		30b	18.2	12.8	16.7
	total major core and Lewis fucosylated *N*-glycans (di Fuc, based on diagnostic ion in MS/MS)	18	5.0	3.7	5.8
		21	3.6	3.0	4.3
		27	10.8	9.2	9.4
		30a	6.3	4.9	4.6
		30b	18.2	12.8	16.7
		36b	6.7	5.8	7.5
		42a	4.1	4.1	5.0
		43a	4.8	4.9	5.7
		54a	2.6	2.6	3.5
		58	2.2	3.4	3.5

Supplemental Fig. 1

D-3

Fuc	*N*-glycans	No.	The relative abundances (%) were calculated by setting the total intensities of major oligomannose *N*-glycans as 100%.		
			Fut8+/+	Fut8+/−	Fut8+/− treated with L−fucose
−	total major oligomannose *N*-glycans	2, 3a, 3b, 4, 5	100	100	100
CoreFuc	most major core fucosylated *N*-glycans (mono Fuc)	35b, 35c, 35d, 35e	79.5	57.9	63.2
	total major core fucosylated *N*-glycans (mono Fuc, based on diagnostic ion in MS/MS)	10, 11, 14a, 17b, 17c, 20b, 21, 26c, 27, 29b, 29c, 30a, 30b, 32c, 33, 35b, 35c, 35d, 35e, 36a, 36b, 39b, 41a, 41c, 42a, 43a, 45b, 46b, 46c, 49a, 49b, 50a, 53a, 54a, 57, 58	358.9	277.8	294.4
CoreFuc + LewisFuc	most major core and Lewis fucosylated *N*-glycans (di Fuc)	30a, 30b	24.5	17.8	21.2
	total major core and Lewis fucosylated *N*-glycans (di Fuc, based on diagnostic ion in MS/MS)	18, 21, 27, 30a, 30b, 36b, 42a, 43a, 54a, 58	64.4	54.4	66.1

Supplemental Fig. 2

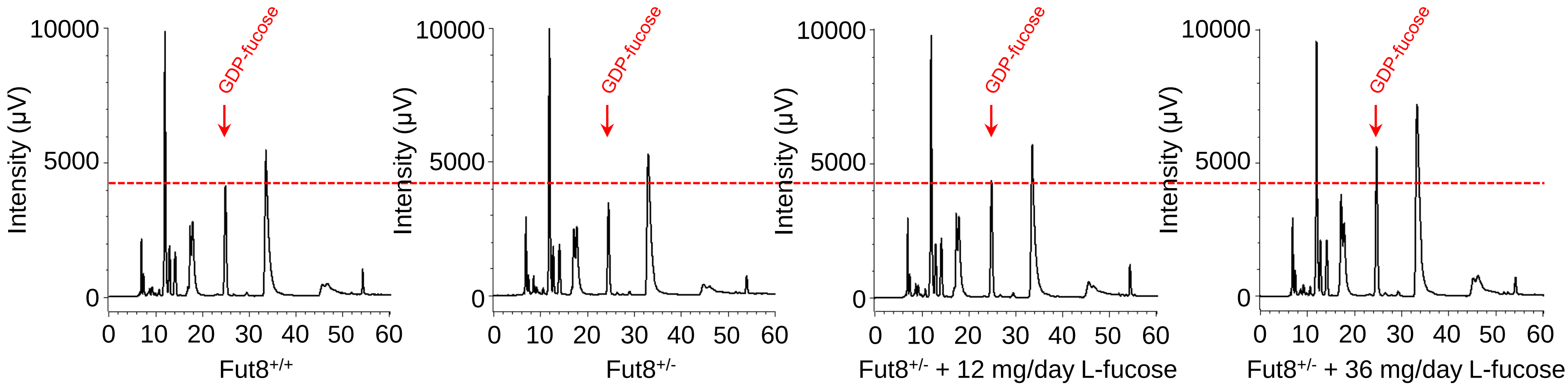

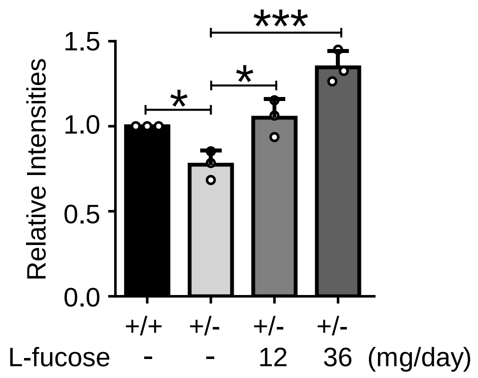

Separation of GDP-fucose in mice brain extracts. The Fut8 mice were treated with equal amounts of PBS or L-fucose (12 or 36 mg/day) for two weeks. The brain tissues (50 mg) were homogenized in ice-cold 70% ethanol (500 μl). All the freeze-dried samples were subjected to ion-pair solid-phase extraction using the Envi-Carb columns. Dried nucleotide sugars were dissolved with 100 μl Milli-Q water and injected 50 μl onto the column. The GDP-fucose peak was confirmed by comparing with the retention time of GDP-fucose standard. All data were shown as the mean ± SD from three independent experiments. The relative intensities showed the intensity of GDP-fucose in each group against that in $Fut8^{+/+}$ mice treated without L-fucose, which was set as 1.0. *$p < 0.05$; ***$p < 0.001$.

Supplemental Fig. 3

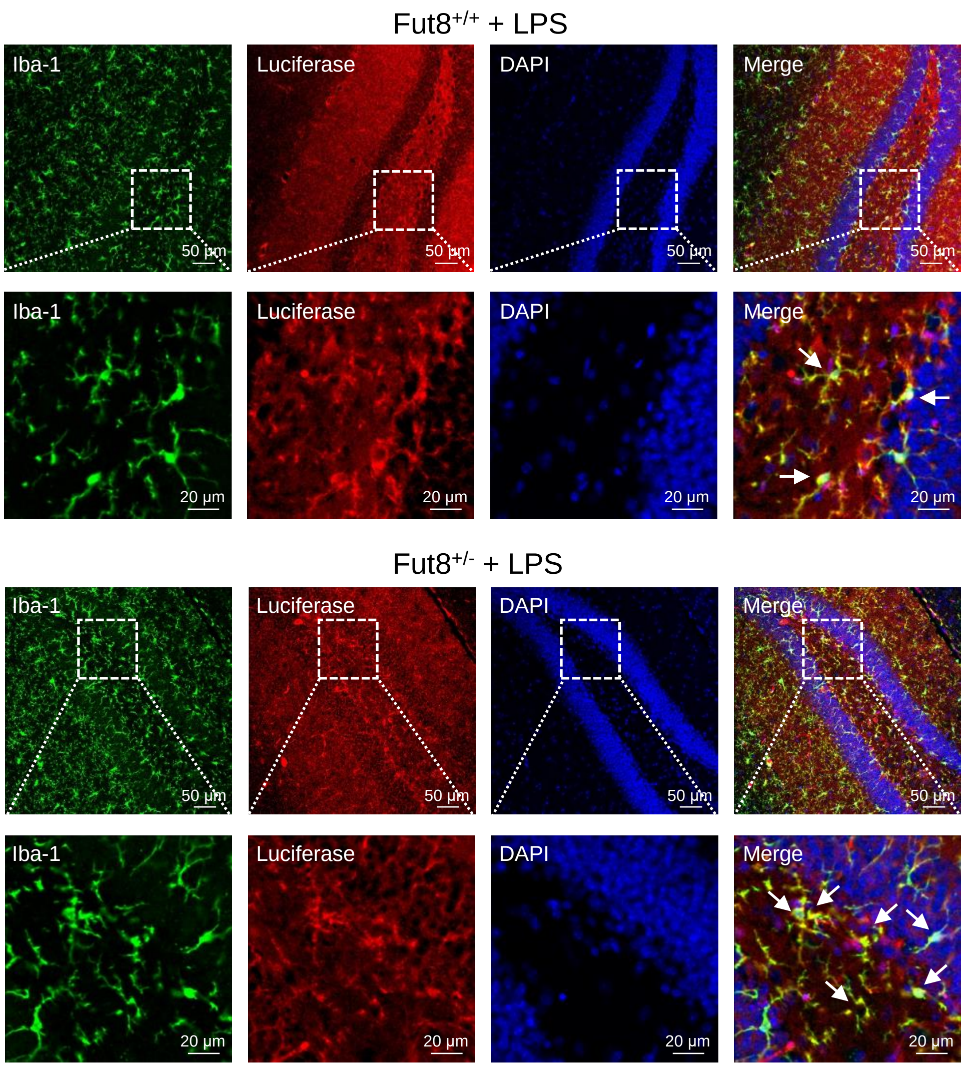

Luciferase was expressed in microglia in the DG of brain tissues. The Fut8::hIL6-Luc mice were intraperitoneally injected with PBS or LPS for 4 h. Representative immunostaining images showed the microglia stained with anti-Iba-1 (green), luciferase stained with anti-luciferase antibody (red) and nucleus stained with DAPI (blue) in the DG of brain tissues. Arrows indicated luciferase (red) in the microglia (green).

Supplemental Fig. 4

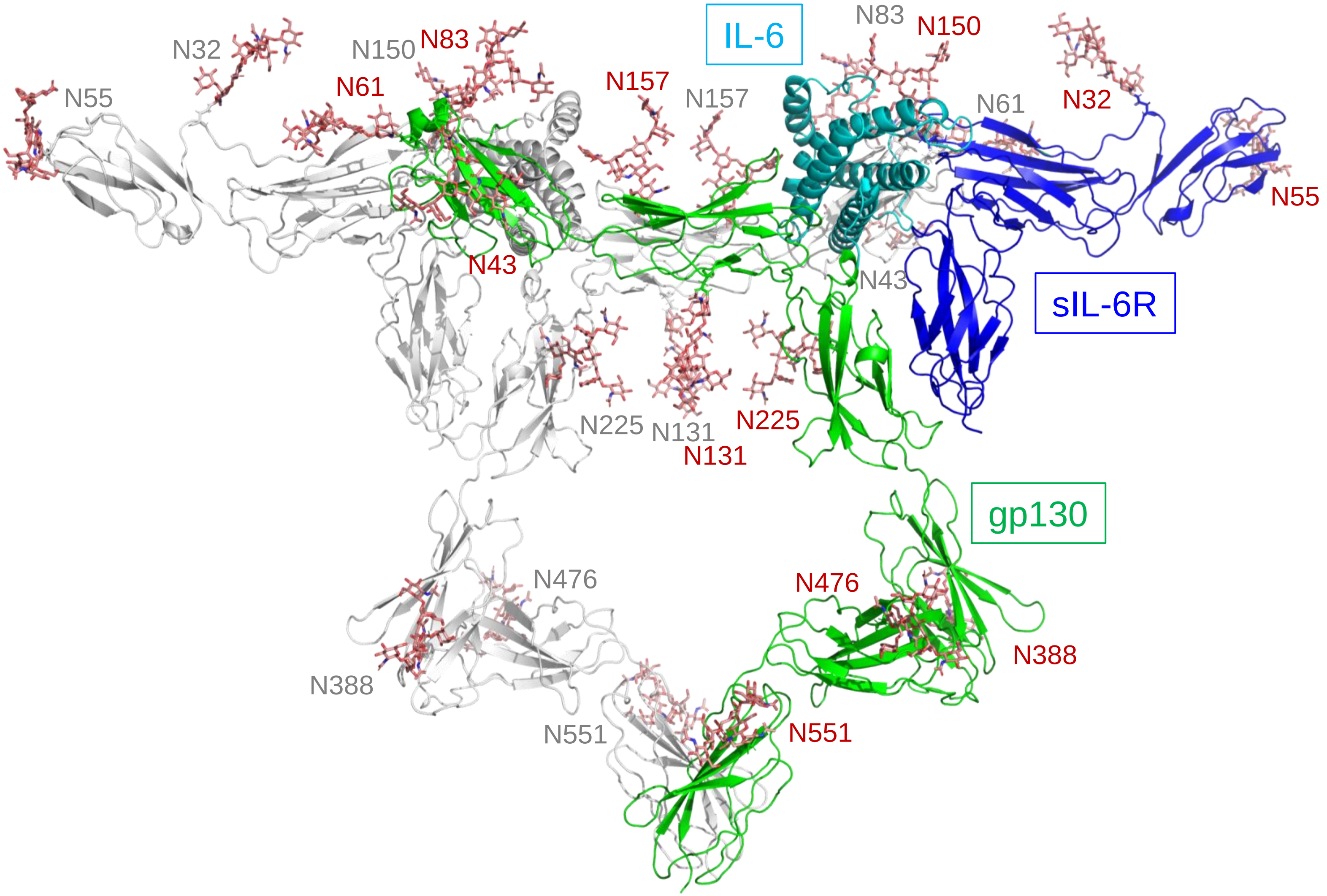

A 3D structural model of *N*-glycosylated IL-6/sIL-6R/gp130 complex. 3D structural model of *N*-glycosylated IL-6/sIL-6R/gp130 complex based on SWISS-MODEL homology modeling using the coordinates of human IL-6/sIL-6R/gp130 complex (PDB ID: 8D82) and Glycan modeler tool in CHARMM-GUI. GlcNAc2Man3GlcNAc2Fuc structure was modeled onto all potential *N*-glycosylation sites. For clarity, half of the complex model is colored: IL-6 is shown in cyan, sIL-6R in blue and gp130 in green with ribbon representation. *N*-Glycans are shown in brown with stick representation. The other half of the complex is shown in gray with ribbon representation. This figure was prepared using the PyMOL software.

Supplemental Fig. 5

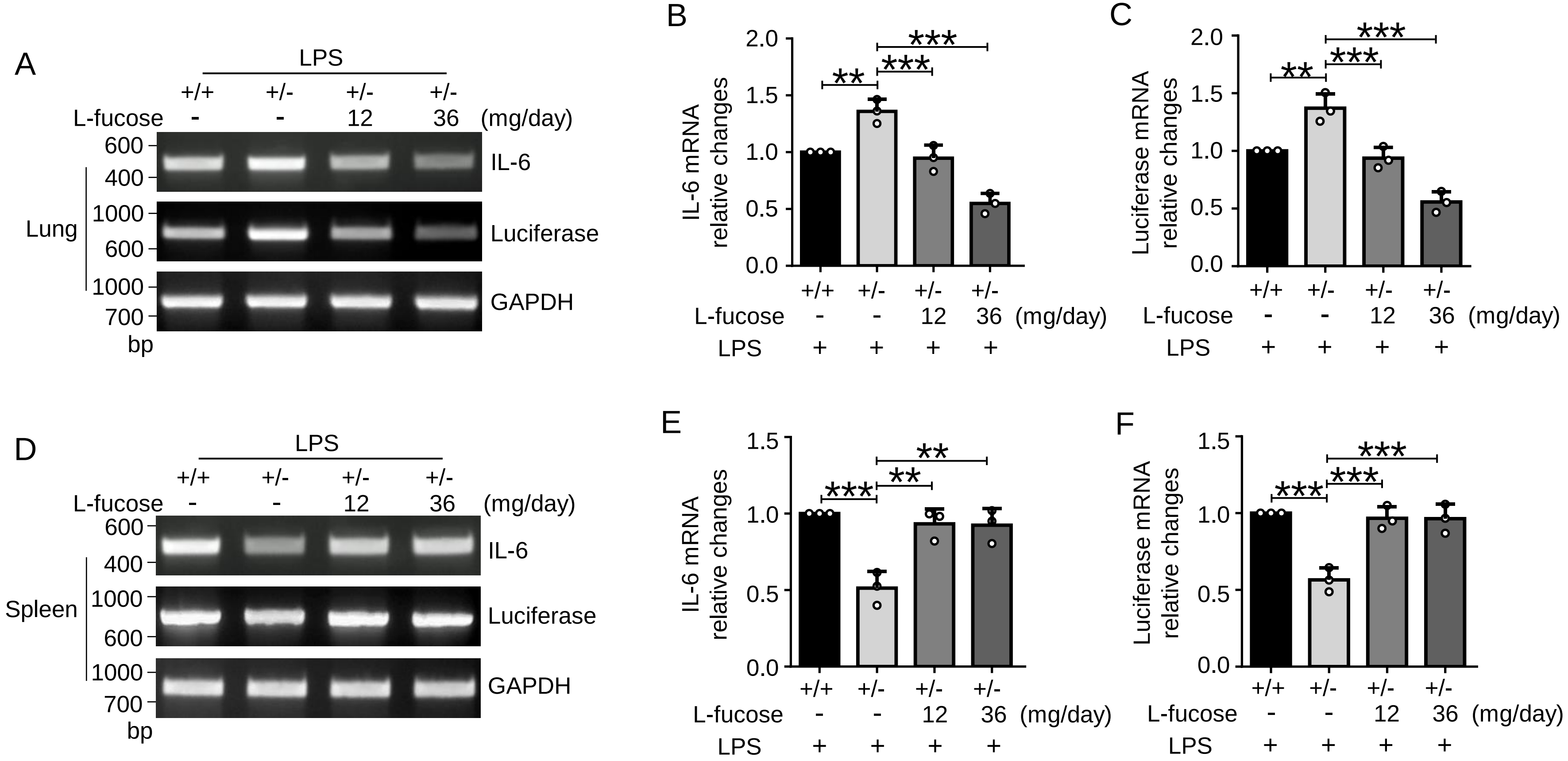

Alteration of IL-6 and luciferase induced by LPS in the lung and spleen tissues. The Fut8::*hIL6-Luc* mice were treated with equal amounts of PBS or L-fucose (12 or 36 mg/day) for two weeks and then intraperitoneally injected with LPS on the 15th day. Post 4 h after intraperitoneal injection, RT-PCR (A, D) and real-time PCR (B, C, E, F) detected the levels of IL-6 and luciferase in the lung (A-C) and spleen tissues (D-F). All data were shown as the mean ± SD from three independent experiments. GAPDH was used as an internal control. Each value was normalized to that of the GAPDH. The value of Fut8$^{+/+}$::*hIL6-Luc* mice treated without L-fucose was set as 1.0. $^{**}p < 0.01$; $^{***}p < 0.001$.
